# How *Streptococcus suis* escapes antibiotic treatments

**DOI:** 10.1186/s13567-022-01111-3

**Published:** 2022-11-12

**Authors:** Cristina Uruén, Carla García, Lorenzo Fraile, Jan Tommassen, Jesús Arenas

**Affiliations:** 1 Unit of Microbiology and Immunology, Faculty of Veterinary, University of Zaragoza, 50017 Zaragoza, Spain; 2 Department of Animal Science, ETSEA, University of Lleida-Agrotecnio, 25198 Lleida, Spain; 3 Department of Molecular Microbiology and Institute of Biomembranes, Utrecht University, 3584 CH Utrecht, The Netherlands

**Keywords:** *Streptococcus suis*, antibiotic resistance, antibiotic tolerance, multidrug resistance, recalcitrance

## Abstract

*Streptococcus suis* is a zoonotic agent that causes sepsis and meningitis in pigs and humans. *S. suis* infections are responsible for large economic losses in pig production. The lack of effective vaccines to prevent the disease has promoted the extensive use of antibiotics worldwide. This has been followed by the emergence of resistance against different classes of antibiotics. The rates of resistance to tetracyclines, lincosamides, and macrolides are extremely high, and resistance has spread worldwide. The genetic origin of *S. suis* resistance is multiple and includes the production of target-modifying and antibiotic-inactivating enzymes and mutations in antibiotic targets. *S. suis* genomes contain traits of horizontal gene transfer. Many mobile genetic elements carry a variety of genes that confer resistance to antibiotics as well as genes for autonomous DNA transfer and, thus, *S. suis* can rapidly acquire multiresistance. In addition, *S. suis* forms microcolonies on host tissues, which are associations of microorganisms that generate tolerance to antibiotics through a variety of mechanisms and favor the exchange of genetic material. Thus, alternatives to currently used antibiotics are highly demanded. A deep understanding of the mechanisms by which *S. suis* becomes resistant or tolerant to antibiotics may help to develop novel molecules or combinations of antimicrobials to fight these infections. Meanwhile, phage therapy and vaccination are promising alternative strategies, which could alleviate disease pressure and, thereby, antibiotic use.

**Supplementary Information:**

The online version contains supplementary material available at 10.1186/s13567-022-01111-3.

## Introduction: the zoonotic pathogen *Streptococcus suis*

*S. suis* resides asymptomatically in the upper respiratory tract, i.e., the tonsils and the nasal cavities, the gut, and the genitals of pigs, as part of the normal microbiota. Piglets can be colonized via horizontal and vertical transmission, caused by nose-to-nose contact and nose-to-vagina contact during farrowing, respectively. The colonization rate can be up to 100%. However, *S. suis* can turn pathogenic when it penetrates mucosal barriers and accesses the bloodstream, joints, and the central nervous system, thereby causing a variety of symptoms such as bacteremia, endocarditis, arthritis, pneumonia, and sudden death [1]. The penetration of the epithelial mucosa and the evasion of innate immune defenses are essential steps for the invasion. For this, *S. suis* produces a large variety of virulence factors, including enzymes, such as proteases and DNases, and toxins, which all contribute to the evasion of the host immune system and to nutrient acquisition within the host [1]. For the invasion process, *S. suis* can additionally take advantage of the depression of mucosal immunity by respiratory viral infections, particularly by swine influenza virus and porcine reproductive and respiratory syndrome virus [2]. Thus, *S. suis* has been considered as a pathobiont [2].

The streptococcal swine disease resulting from *S. suis* infection is a major cause of mortality and economic losses in the pig production industry worldwide. It has been estimated that the incidence ranges ranged from 5 to 20%, but this largely varies between regions and farms. Notably, the disease is a leading cause of mortality in piglets aged 4–12 weeks, but it may also affect both younger and older pigs. About 70% of the cases where the infection reaches the nervous system end in death. Much of the economic losses are attributed to mortality, pig management and attempts to control infection, but the disease can also reduce weight gain and raise production costs.

Historically, antibiotics have been used to prevent *S. suis* cases, but this practice is nowadays prohibited in many countries. Also, vaccination is used to prevent infection, but its efficacy is limited. Only bacterins are applied in the field for immunization of piglets or sows. Bacterins are suspensions of whole killed bacteria prepared from invasive clones collected in certain farms. The protection provided by bacterins is strain specific and often unpredictable [3]. The main drawbacks associated with bacterins are: (i) high diversity of antigens produced by *S. suis*, (ii) antigenic variability of surface-exposed structures, and (iii) loss of the tertiary structure of many antigens during cell inactivation required for bacterin production. Therefore, bacterins are not universal and have limited effectiveness in preventing *S. suis* outbreaks. In this context, treatment of the disease is mainly based on antibiotic therapies combined with the use of bacterins to avoid the expansion of the disease, while prevention is limited to managing environmental conditions and, only in particular farms affected by certain clones, to the use of bacterins. The lack of an effective and universal vaccine formula to prevent or reduce the appearance of *S. suis* infections together with the high incidence and mortality of the infection has provoked the exhaustive use of antibiotics for a long time. In addition, *S. suis* is, as a commensal bacterium, exposed to antibiotics used for growth promotion, prophylaxis, and the treatment of other infectious diseases. All factors together have created a good scenario for the emergence of antimicrobial-resistant (AMR) *S. suis* isolates.

## *S. suis* is a widespread superbug

**Figure 1:**
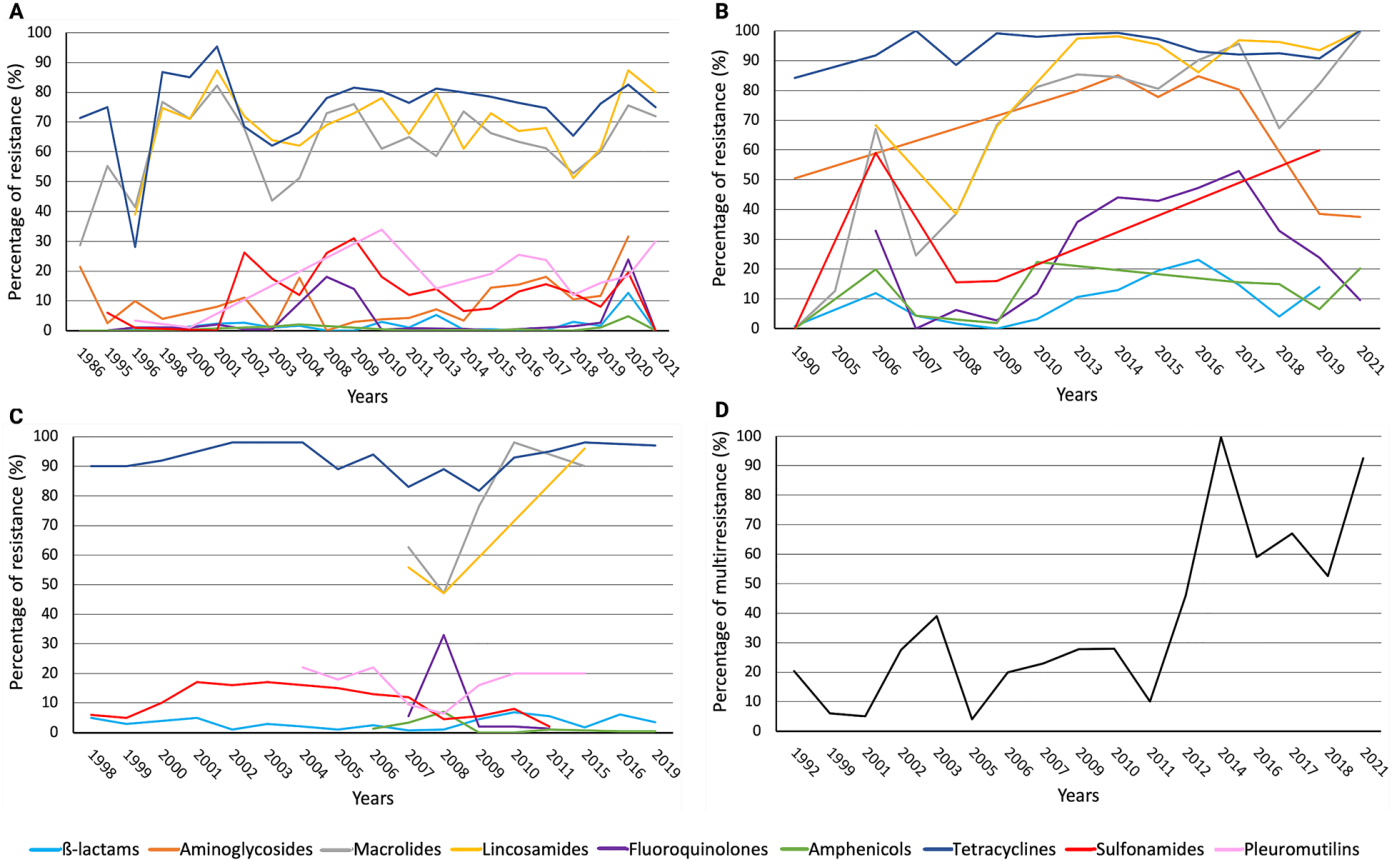
**Historical development of resistance of *S. suis* to different antibiotic classes in A Europe, B Asia, and C America (North and South America).** The graphs include data reported between 1987 and 2021 in scientific articles published in National Library of Medicine under search criterium ¨antimicrobial resistance and *Streptococcus suis*¨, and the antibiotic surveillance data from Denmark (DANMAP) and France (RESAPATH). For panel **A**, a total of 19 articles and 18 reports (DANMAP, RESAPATH) were used, and totals of 13 and 8 articles were used for panels **B** and **C**, respectively. For simplicity, the averages of the percentage of resistance to antibiotics of the same class were calculated for each report. Please note that not all the surveillance systems are using the same Epidemiological cut-off value ECOFFs; we followed the criteria used by authors. The evolution of the multidrug-resistance rate worldwide is depicted in **D**, taking into consideration a total of 20 scientific articles. Multiresistance was considered when an isolate was resistant to at least three antibiotics of different classes as declared by authors. Also, the average of the antimicrobial-resistance rate of different reports in the same year was used. The X-axes show the year of isolation.

Antibiotics used to treat *S. suis* infection are multiple, and they are from different classes, including β-lactams, aminoglycosides (usually combined with β-lactams), amphenicols, and fluoroquinolones. The pattern of antibiotic usage varies between countries, regions, and even farms, which largely influences the AMR profile of *S. suis*. AMR in *S. suis* was first reported around the 80 s of the previous century, and since then, the AMR rates increased over time globally. Figure 1 shows the historical development of AMR rates for different classes of antibiotics in *S. suis* isolates from Europe, Asia, and America, compiled by using publications and reports from the last 20 years. For simplicity, the rates of resistance were calculated for each class of antibiotics (including different antibiotics of the same family) even though, in some studies, resistance varied between different antibiotics of the same class. In Additional file 1, information regarding the methods used to measure antibiotic resistance and tolerance in *S. suis* is expanded.

In Europe, the highest AMR rates were reported for lincosamides, macrolides, and tetracyclines, as is evident from numerous reports since 1998. Lincosamides, including lincomycin and clindamycin, together with streptogramin B and macrolides, often showed cross resistance due to their common mechanisms of action (discussed in the next section). They have been used largely in food production animals, particularly in intensive pig farming, to treat a variety of infectious diseases. This may explain the high resistance rates registered in *S. suis* isolates in last two decades (Figure 1A). For example, tylosin (Tylan) has been extensively used as growth promoter, and this could be related to high macrolide resistance rates observed. Also, notable differences were reported between countries over time. For example, macrolide (erythromycin)-resistance rates ranged from 29% in Norway in 1986 [4] to 56% in Denmark in 2020 [5], and lincosamide (lincomycin)-resistance levels were 40% in Denmark in 1995 [6], 61% in France in 2019 [7], and up to 87% in Spain in 2021 [8]. Together with lincosamides, tetracyclines were also among the most widely used antibiotics in the European pig industry because of their broad spectrum. As a consequence, many European countries reported tetracycline-resistance rates higher than 60% since the end of the 90 s, for example 68% in England in 2004 [9], 73% in France in 2011 [10], and 88% in Sweden in 2019 [11].

Because of the high resistance rates, the aforementioned antibiotics have been replaced by others, such as β-lactams of which the resistance rates have persisted low over time (Figure 1A). For example, resistance rates of less than 10% to β-lactams were reported in Denmark (1995) [6], Spain (2000) [12], Poland (2004) [9], The Netherlands (2014) [13], and, more recently, in Czech Republic (2020) [14]. These data indicate that the development of β-lactam resistance is rather modest in *S. suis*. Also, quinolones have been used as an alternative to tetracyclines. Reports indicate resistance rates of less than 10% in the last two decades in several European countries but with few exceptions (Figure 1A). Good examples are Belgium (2000) [15], The Netherlands (2014) [13], and Sweden (2019) [11], where the resistance rates remained well below 10% in this period. However, the high rates reported in France (18% in 2008) [16] and Spain (47% in 2020) [8] suggest that fluoroquinolone resistance may become problematic in the future. Other antibiotics used to treat *S. suis* infections in Europe were aminoglycosides (often combined with β-lactams), amphenicols, sulfonamides, and pleuromutilins. Reports have notified low or medium AMR rates. For example, aminoglycoside-resistance rates lower than 10% were continuously reported in France in 2002 [17], 2009 [18], 2014 [19], and 2017 [20]), but high rates were reported in Denmark in 2016 (up to 41%) [21], 2018 (up to 36%) [22], and in 2020 (44%) [5]. Sulfonamide-resistance rates showed large variation between countries in the same time period, ranging from 3% in The Netherlands in 2016 [13] to 21% in France in 2017 [20] or up to 94% (sulfadimethoxine) in Spain in 2021 [8]. For amphenicols, AMR rates remained very low in the last two decades, e.g*.* in Belgium (2013) [23], Denmark (2016) [21], and Czech Republic (2021) [14]. In contrast, AMR rates to pleuromutilins have been moderate, showing an increasing trend in recent years, with the highest reported rates in Denmark (24% in 2017) [24] and Czech Republic (31% in 2021) [14]. Overall, AMR rates to most antibiotics vary considerably between European countries. This could be related to differences in pig production, growth systems, or the political regulations concerning the usage of antibiotics. For instance, The Netherlands and France were big antibiotic consumers, followed by the United Kingdom, Czech Republic, Switzerland, Germany, and Denmark. In contrast, antibiotics were less frequently used in Finland, Sweden, and Norway, due to the less intensive pig industry [25].

In Asia, the AMR rates for tetracyclines, lincosamides, and macrolides are extremely high (Figure 1B), even higher than in Europe (Figure 1A). In general, resistance to tetracyclines is around 95%, showing little variation over the last two decades. Examples are 92% in China in 2005–2007 [26], 98% in Korea in 2010 [27], or up to 92% in Thailand in 2019 [28]. Notably, tetracycline-resistant *S. suis* isolates were common in both diseased and healthy pigs [26] as well as in diseased humans [29]. Many reports revealed increasing rates of resistance to lincosamides and macrolides since 2012. This is illustrated in reports from China notifying rates of resistance for macrolides in *S. suis* from up to 35% in isolates from the period 2005–2010 [30], to 68% in isolates from 2008 to 2010 [31], 87% in isolates from 2013 [32], and up to 97% in isolates from 2017 [33]. A concomitant increase in the rates of resistance to lincosamides was reported from 39% in the period 2005–2012 [34], to 98% in 2008–2010 [35], 96% in 2015 [32], and up to 100% in 2021 [36]. Recent studies in other Asian countries, such as Thailand (2019) [28] and India (2021) [37], notified similar AMR profiles. AMR rates for sulfonamides, aminoglycosides, and fluoroquinolones, which were moderate in Europe, ranged from medium to high in Asia (compare panels A and B in Figure 1). The rates varied between countries, but, in general, there was a notable increase since 2010 (Figure 1B). Thus, the rates of resistance for sulfonamides (trimethoprim/sulfamethoxazole) in earlier reports were 0% in Japan (isolates from 1987 to 1996) [38] or 16% in China (isolates from 2005 to 2012) [34], and they were higher after 2010, for instance 60% in Thailand in 2019 [28]. The resistance rates for aminoglycosides are higher than for sulfonamides with, e.g., reported rates in China ranging from 62% in 2012 [35] to 87% in 2019 [32], indicating an increasing trend. The reported resistance rates for fluoroquinolones remained below 40%, but they are also increasing, e.g., China showed 6% resistance in 2015 [34] and up to 37% in 2021 [39]. β-Lactams still showed the lowest resistance rates, although they were higher than in Europe, reaching values still below 15% for instance in China in 2014 [30] and in 2019 [32]. Yet, large differences between various β-lactam antibiotics were detected. In a study in Thailand (2019), AMR rates were found of around 20% for penicillin and ampicillin, 5% for cefotaxime and ceftiofur, and up to 35% for cephalexin [28]. For amphenicols, low resistance rates were reported, e.g., of 4% in China in 2015 [30] and in 2020 [33]. Curiously, a notable drop in resistance rates for aminoglycosides and fluoroquinolones was observed from 2018 on (Figure 1B). Altogether, these data show, in general, higher AMR rates in Asia than in Europe. This could be due to the massive use of antibiotics as growth promoters and the lack of regulation for the sales of antibiotics, which might have generated a selective pressure for resistance. Both practices also occurred in Europe, but to a lesser extent. For example, the use of antibiotics as growth promoters was banned at an early stage. Sweden and Denmark were the first countries that banished the use of antibiotics as growth promoters in 1986 and 2000, respectively, and this was followed by a general prohibition in the whole European Union (EU) in 2006. However, whereas, for example, Japan, Taiwan, and Hong Kong have similar restrictions, most Asian countries still use antimicrobials for this purpose with exceptions only for some specific antibiotics [40]. Few reports from Oceania revealed overall low resistance rates for β-lactams, amphenicols, fluoroquinolones, and sulfonamides, moderate rates for macrolides, and the highest rates for tetracyclines (almost 100%), with some differences between reports [41, 42]. In general, for most antibiotics, AMR rates were considerably lower in Oceania than in Asia.

In North America, AMR rates for tetracyclines, lincosamides, and macrolides were the highest, being > 80% for tetracyclines and > 50% for aminoglycosides and lincosamides in 2007. An example is Canada reporting rates of 89% and 97% for tetracyclines in *S. suis* isolates from 2005 [43] and 2019 [44], respectively, of 77% and 90% for macrolides (erythromycin) in isolates from 2007 to 2001 [45] and from 2013 to 2018 [46], respectively, and of 96% for lincosamides (clindamycin) in isolates from 2013 to 2018 [46]. In contrast, resistance rates for other antibiotic classes (β-lactams and amphenicols) have remained < 10% up to date (Figure 1C) [45, 47], while, in general, moderate rates (up to 25%) were reported for sulfonamides and pleuromutilins during the last two decades (Figure 1C) [43, 46]. Few data have been reported about aminoglycosides and fluoroquinolones, whose AMR rates varied between the different antibiotic types [48] over time [45, 48]. Very little data have been reported in South America. Low-medium AMR rates for β-lactams (up to 18%) and amphenicols (up to 14%) were reported, while higher rates for fluoroquinolones (up to 77%), aminoglycosides (up to 50%), sulfonamides (up to 100%), lincosamides (85%), macrolides (up to 66%), and tetracyclines (98%) were described in Brazil [49, 50].

Altogether, resistance against antibiotics in *S. suis* is a worldwide problem and, particularly, resistance rates are very high for tetracyclines, macrolides, and lincosamides. In contrast, resistance rates for β-lactams, fluoroquinolones, and amphenicols remain low, but recent reports suggest an increasing trend. This has been associated with the occurrence of multidrug resistance, i.e.*,* simultaneous resistance to antimicrobials of at least three different classes, which increased over time (Figure 1D). Alarmingly, multidrug-resistant isolates have been recovered from diseased humans as reported by Hoa et al. [51], who showed multidrug resistance to tetracycline, erythromycin, and chloramphenicol in *S. suis*-infected patients and a significant increase in multidrug resistance from 6% in 1999 to 23% in 2006 and 2007. These data point out that *S. suis* is a globally disseminated bacterial ¨superbug¨.

## Genetic basis of *S. suis* resistance to antibiotics

**Figure 2:**
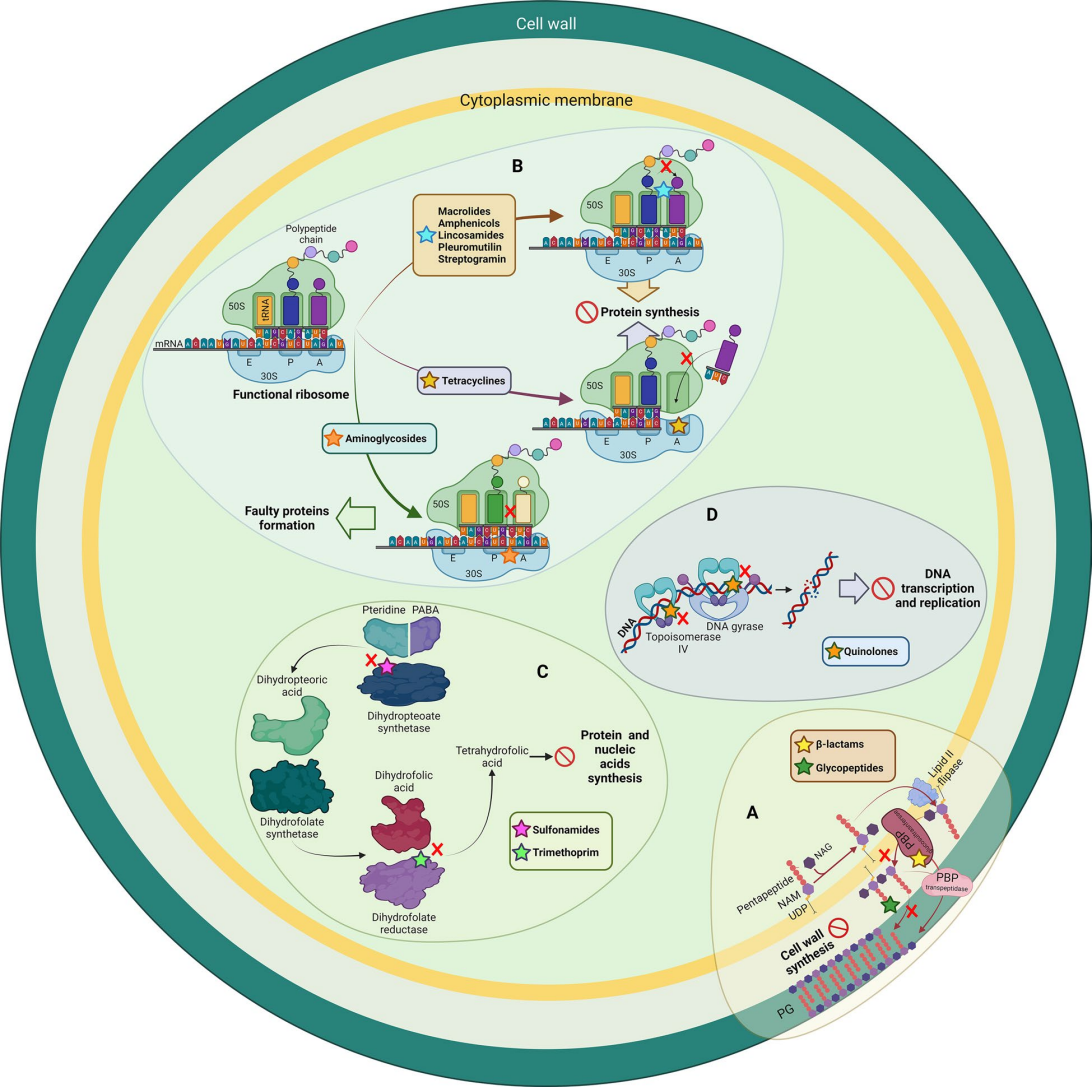
**Overview of the mode of action of antibiotics used to treat *S. suis* infections.** According to its target site, antibiotics can be classified as **A** inhibitors of peptidoglycan (PG) synthesis by blocking penicillin-binding proteins (PBPs) (β-lactams) or binding D-Ala-D-Ala in the peptide chain of the PG precursor lipid II (glycopeptides), **B** inhibitors of ribosome function that bind to the ribosome 50S (macrolides, amphenicols, lincosamides, and pleuromutilins) or 30S (tetracyclines and aminoglycosides) subunits, **C** inhibitors of folic acid synthesis including sulfonamides and trimethoprim, and **D** inhibitors of DNA transcription and replication by interfering with DNA gyrase or topoisomerase IV (quinolones). In **A**, the process of the synthesis and transport of PG precursors is indicated. The 30S and 50S ribosome subunits and the A, P, and E sites are indicated in **B**. The A site is the binding site for charged tRNA molecules during protein synthesis. Then, the tRNA moves to the P site and its cargo is then linked to the growing polypeptide chain. Thereafter, the tRNA is moved to the E site for exit. The route for folate synthesis is shown in **C**. In **D**, the requirement for DNA gyrase and topoisomerase IV in DNA transcription and replication processes is shown. Antibiotics are indicated with colored stars. A red circle indicates resulting inhibition of the cellular process. Abbreviations: PABA, *p*-aminobenzoic acid; pteridine, 7,8-dihydro-6-hydroxymethylpterin-pyrophosphate; UDP, uridine diphosphate.

The mode of action of the above-mentioned antibiotics is class-dependent, as schematized in Figure 2. Overall, antibiotics obstruct vital biological processes, such as cell-wall synthesis, protein synthesis, or DNA replication, mainly by interfering with the activity of specific enzymes. Different classes of antibiotics can share similar targets but make use of different mechanisms. To counteract antibiotic activity, *S. suis* has developed various mechanisms of resistance that generally fall into four categories: (i) target mutation, often preventing the binding of an antibiotic to its target, (ii*)* enzymatic target modification, (iii) antibiotic modification, comprising degradation of the antibiotic or its modification, thereby preventing target binding, and (iv) export of antibiotics out of the cell by increased production of efflux pumps. AMR genes of *S. suis* and resistance and tolerance mechanisms have been reviewed previously [29, 52, 53]; here, we have updated the AMR list in Table 1. Often, accumulation of different genes and mechanisms enhance resistance. Below, the mode of action of the antibiotics used to treat *S. suis* infections and the reported resistance and tolerance mechanisms are discussed.

**Table 1:** **Genes related to antimicrobial resistance in** ***S. suis***

Gene names^a^	Relevant characteristics^b^	Reference(s)
β-Lactams		
*Target mutation*		
pbp1a^*1^	PBP1a (A365S, P409T, V412F, E447A, N459D, H464Y, S477G, D479Q, A493T, K522Q, K255Q, N550D, S578A)	[65]
*pbp2b*/*pen*(A)^*1^	PBP2b (K479T/A, D512E/Q/K/A, K513E/D, T515S)	[62, 65]
pbp2X^*1^	PBP2x (M437L, S445T, T467S, Y525F, T551S, N569Q, L594Y/F, V596G)	[62, 65]
*Mutation in non-targeted genes*		
mraY^*1^	Phospho-N-acetylmuramoyl-pentapeptide-transferase (M6I/L and either A4S/T or G8S)	[62]
Macrolides		
*Target modification*		
*erm*(A)^*2^	23S rRNA methyltransferase	[236]
*erm*(B)^*2^	23S rRNA methyltransferase	[236]
*erm*(C)^*2^	23S rRNA methyltransferase	[88]
*erm*(G)^*2^	23S rRNA methyltransferase	[62]
*erm*(T)	23S rRNA methyltransferase	[62]
cfr^*2^	23S rRNA methyltransferase	[84]
*Antibiotic modification*		
*mph*(B)	Macrolide 2'-phosphotransferase	[39]
*mph*(C)	Macrolide 2'-phosphotransferase	[88]
*Efflux pump*		
*mef*(A/E) */ mel*(D) (*a.k.a*. *msr*(D)) ^*3^	MFS transporter / ABC F RPP	[31, 39, 88, 89]
Lincosamides		
*Antibiotic modification*		
*lnu*(A)	Lincosamide nucleotidyltransferase (adenylation)	[236]
*lnu*(B)	Lincosamide nucleotidyltransferase (adenylation)	[39, 237]
*lnu*(C)	Lincosamide nucleotidyltransferase (adenylation)	[145]
*lnu*(E)	Lincosamide nucleotidyltransferase (adenylation)	[238]
*Target modification*		
*erm*(A)^*2^	23S rRNA methyltransferase	[236]
*erm*(B)^*2^	23S rRNA methyltransferase	[236]
*erm*(C)^*2^	23S rRNA methyltransferase	[88]
*erm*(G)^*2^	23S rRNA methyltransferase	[62]
*erm*(T)	23S rRNA methyltransferase	[239]
cfr^*2^	Ribosome methyltransferase	[84]
*lsa*(E)^*2^	ABC-F RPP	[237]
*vga*(F)^*2^	ABC-F RPP	[62]
optrA^*2^	ABC-F RPP	[97, 115]
Pleuromutilin		
*Target modification*		
*lsa*(E)^*2^	ABC-F RPP	[145, 237]
*vga*(F)^*2^	ABC-F RPP	[62]
Amphenicols		
Antibiotic *modification*		
*cat*(A)	Chloramphenicol acetyltransferase	[97, 143]
*Target modification*		
optrA^*2^	ABC-F RPP	[33, 83, 97, 101, 115, 117]
cfr^*2^	Ribosome methylase	[84]
*Efflux pump*		
*fexA*	MFS transporter	[84]
Tetracyclines		
*Target modification*		
*tet*(M) [*tet*(M1), *tet*(M2), *tet*(M3)]^*4^	RPP antibiotic competition	[31, 51, 62]
*tet*(O) (*tet*O1)^*5^	RPP, antibiotic competition	[31, 51]
*tet*(O/32/O)^*6^	RPP, antibiotic competition	[31]
*tet*(O/W/32/O)^*6^	RPP, antibiotic competition	[31]
*tet*(S)	RPP, antibiotic competition	[31]
*tet*(W)	RPP, antibiotic competition	[31, 51]
*tet*(44)	RPP, antibiotic competition	[62]
*Efflux pump*		
*tet*(B)	MFS transporter	[240]
*tet*(K)	MFS transporter	[39]
*tet*(L)	MFS transporter	[31, 51]
*tet*(40)	MFS transporter	[31]
Trimethoprim		
*Target modification*		
*dfrF*	Trimethoprim-resistant dihydrofolate reductase	[62]
*dfrK*	Trimethoprim-resistant dihydrofolate reductase	[62]
*Target mutation*		
dhfr^*1^	Dihydrofolate reductase (I102L)	[62]
*dhfr* promoter^*1^	Dihydrofolate reductase (A5G)	[62]
Aminoglycosides^*7^		
*Antibiotic modification*		
* sat4*	Streptothricin N-acetyltransferase	[116, 119]
*ant1*	Aminoglycoside O-nucleotidyltransferase ANT	[62]
*ant(4’)-Ib*	Aminoglycoside O-nucleotidyltransferase ANT	[62]
ant(6)-Ia a.k.a. ant6, aadE (aadE1, aadE2)^*8^	Aminoglycoside O-nucleotidyltransferase ANT	[62, 89, 115, 116, 148]
*ant(6)-Ib*	Aminoglycoside 6'-N-acetyltransferase	[62]
* ant(9’)-Ia, a.k.a. aad9*	Aminoglycoside nucleotidyltransferase	[62, 116]
*aph(6)-Ia*	Aminoglycoside O-phosphotransferase	[97]
*aph(3’)-IIIa a.k.a. aphA3*	Aminoglycoside O-phosphotransferase	[116, 121]
aac(6’)-aph(2’’) a.k.a. aacA, aphD^*9^	Aminoglycoside N-acetyltransferase—O-phosphotransferase	[97, 115]
aac(6’)-Ie-aph(2’’)-Ia^*9^	Aminoglycoside N-acetyltransferase—O-phosphotransferase	[116]
Glycopeptides^*10^		
* Target modification*		
*vanG*	D-Ala-D-Ser ligase	[117, 121]
*vanT*	D-ser producing serine racemase	[121]
*vanXY*	D-Ala-D-Ala dipeptidase / D-Ala-D-Ala carboxypeptidase	[117, 121]
*vanZ*	Putatively alters the binding of (lipo)glycopeptides to cells	[122]
Quinolones		
*Target mutation*		
parC^*1^	Topoisomerase IV (Ser79, Asp83)	[88, 131, 241]
parE^*1^	Topoisomerase IV (Pro278)	[131]
gyrA^*1^	DNA gyrase (Ser81, Glu85)	[62, 88, 131, 241]
gyrB^*1^	DNA gyrase (Glu354, Asp315)	[62, 131]
*Efflux pump*		
*satAB*	ABC transporter	[135]

^a *1^ Mutated target gene. ^*2^ AMR genes conferring resistance to several antibiotics. ^*3^ The *mef* variants are not distinguished here. ^*4^ Three *tet*(M) variants, *tet*(M1), *tet*(M2) and *tet*(M3), were identified in Hadjirin, et al., [62]. ^*5^ A novel *tet*(O) variant designated *tet*(O1) was recently identified by Hadjirin, et al., [62]. ^*6^ These are mosaic genes consisting of fragments of the *tet* genes mentioned in the order of 5’ to 3’. ^*7^ There are two main nomenclatures in use for aminoglycoside-modifying enzymes (reviewed in Ramirez and Tolmasky [114]). Shortly, in the proposal of Shaw et al. [242], genes are identified by a three letter identifier followed by the site of modification in parentheses, then a Roman numeral indicating the resistance profile and, in some cases, a letter when multiple enzymes exist that modify the same position. In the proposal of Novick et al. [243], the genes are designated with three letters followed by a capital letter that identifies the site of modification and then a number to provide a unique identifier to different genes. The nomenclatures are interchangeably used in the literature, and both are provided in the table. ^*8^ Two variants, *aadE*1 and *aadE*2, were recently reported by Hadjirin et al. [62]. ^*9^ AAC(6´) enzymes can exist as fusion proteins linked to APH, ANT, or a different AAC, occupying the N- or C-terminal region and resulting in bifunctional enzymes. ^*10^ There are six different molecular resistance types (named Van-A to Van-G) that confer resistance to glycopeptides by modifying the peptidoglycan structure and that can be organized in gene clusters. Reported individual genes are indicated. ^**b**^ Functions of the gene products, and, when appropriate, the substitutions in antibiotic targets reported in clinical antibiotic^−^resistant isolates are shown. Abbreviations: ABC, ATP-binding cassette; MFS, major facilitator family; PBP, penicillin-binding protein; RPP, ribosome protection protein.

### Resistance to β-lactams

**Figure 3:**
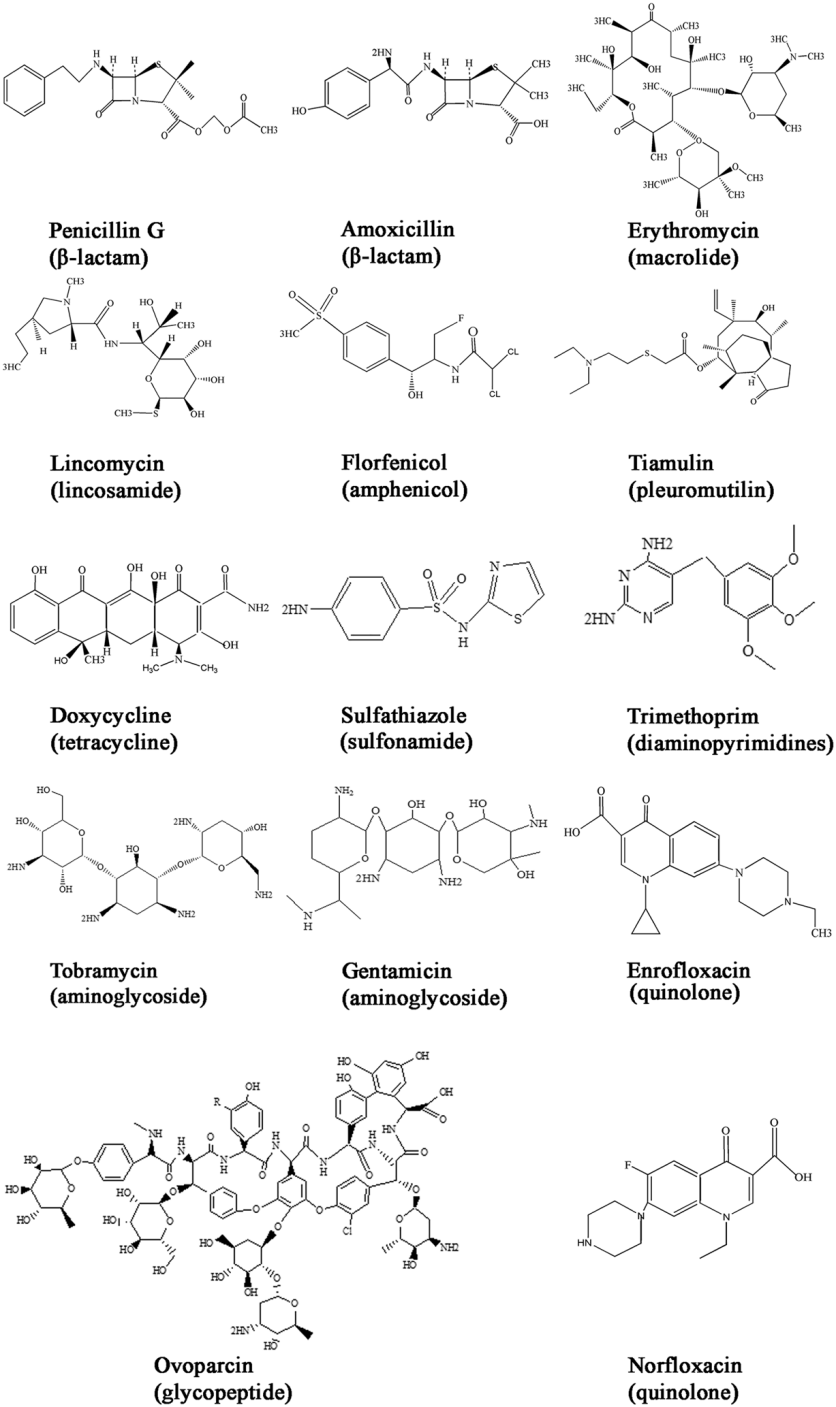
**Chemical structure of diverse antibiotics**.

β-lactams comprise several large families of antibiotics, including penicillins. Penicillins share a thiazolidine ring attached to a β-lactam ring and a side chain (Figure 3). There are five classes of penicillins, including natural penicillins (penicillin G and V), penicillinase-resistant penicillins (methicillin), aminopenicillins (ampicillin), extended-spectrum penicillins (carbenicillin), and aminopenicillin/β-lactamase inhibitor combinations (amoxicillin/clavulanic acid). In addition, cephalosporins, carbapenems, and monobactams have a β-lactam ring and, therefore, they are also classified as β-lactams. Amoxicillin and other penicillins are nowadays broadly used to treat *S. suis* infections. β-Lactams blocks the activity of enzymes involved in cell-wall synthesis by binding of the β-lactam ring to the active site of the penicillin-binding proteins (PBPs) (Figure 2A) at the DD-transpeptidase domain [54]. As a result, new peptidoglycan (PG) precursors can be incorporated into the PG, as the antibiotics inhibit the transpeptidation reaction that establishes crosslinks between the PG polymers (Figure 2A), thus preventing the formation of a rigid cell wall and resulting in cell death. *S. suis*, as other streptococci, is generally susceptible to β-lactam antibiotics (Figure 1A–C).

There are three main mechanisms of resistance to β-lactam antibiotics comprising (i) reduced access to PBPs, (ii) reduced PBP binding affinity, and (iii) destruction of the antibiotic through β-lactamases [55]. In Gram-positive bacteria, loss of β-lactam sensitivity is mostly driven by alterations in the transpeptidase domains of PBPs that decrease the affinity of the enzyme for β-lactams. There are several *pbp* genes in streptococci [56], coding for PBP1a, PBP1b, PBP2a, PBP2b, PBP2x, and PBP3. Three of them are critical for β-lactam susceptibility or resistance, i.e., PBP1a, PBP2b, and PBP2x. This matches with the essential role of some of these enzymes. PBP2b and PBP2x are members of elongosome and the divisome complex, respectively, and are essential for bacterial survival. PBP1a, PBP1b and PBP2a are redundant, and they can be depleted individually. PBP3 and PBP1a are dispensable, but their depletion causes morphological changes and loss of fitness. This is also the case with the double mutants *pbp1a/pbp1b* and *pbp1b/pbp2a*, but, in contrast, a mutant lacking *pbp1a* and *pbp2a* is not viable. As PBP1a, PBP2b, and PBP2x are key targets for β-lactam antibiotics [56], they are often examined to assess the genetic origin of resistance to these drugs. Resistance to β-lactams in streptococci is primarily caused by mutations within the *pbp2x* and *pbp2b* genes [57]. These mutations confer a modest level of resistance, while they can confer high-level resistance in combination with mutations within *pbp1a* [58, 59]. Interestingly, a sequential order of mutations has been observed in several streptococcal species. In *S. pneumoniae*, mutations within *pbp2x* and/or *pbp2b,* depending on the specific antibiotic used*,* are the first event to occur [60], and they are followed by mutations in *pbp1a*. It has been proposed that the secondary mutations, besides increasing antibiotic resistance, are selected to compensate for a fitness cost resulting from the *pbp2b* mutations [61]. Recent work in *S. suis* exploring the origin of β-lactam resistance also reached similar conclusions [62]. Mutations occur first in *pbp2b* and then in *pbp2x* (Table 1). Analysis of β-lactam-resistant mutants of *S. pneumoniae* selected in the laboratory revealed that point mutations in *pbp* genes suffice to confer β-lactam resistance, while analysis of resistant clinical isolates showed a mosaic of sequences that may diverge in about 20% compared to susceptible isolates (reviewed in Hakenbeck et al. [63]). It is entirely possible that the resistance alleles contain many substitutions that are compensatory mutations that alleviate the fitness costs of the resistance mutations. Remarkably, a study exploring 2528 invasive *S. pneumoniae* isolates related β-lactam resistance levels to sequence signatures in the *pbp1a*, *pbp2b*, and *pbp2x* genes [64], which has generated a classification system of PBP types for pneumococci. Thus, PBP types identified based on *pbp* gene sequences are related to particular Minimal Inhibitory Concentrations (further described in Additional file 1). Such a system has not yet been proposed for *S. suis,* but it could help to classify the increasing β-lactam-resistant isolates (Figure 1A–C).

Apart from mutations in *pbp1a*, *pbp2b*, and *pbp2x*, mutations in *pbp1b*, *pbp2a*, and *pbp3* have been described in 10 β-lactam-resistant *S. suis* isolates [65]. As the corresponding PBPs are no key targets for β-lactam antibiotics, the contribution of these mutations to β-lactam resistance remains to be clarified. Presumably, they contribute to compensate for the fitness cost of mutations within *pbp1a*, *pbp2*x, and/or *pbp2b*. In addition, mutations in the *mraY* gene, which codes for the enzyme that transfers N-acetylmuramyl-pentapeptide-1-phosphate to undecaprenyl phosphate to generate the PG precursor lipid I, have been detected in 62 penicillin-resistant *S. suis* isolates [62] (Table 1). As MraY is not a target for the β-lactams, probably also these mutations are compensatory mutations. Besides, mutations in the two-component regulatory system CiaRH [66], the glycosyltransferase CpoA [67], and the cell-wall muropeptide-branching enzyme MurM [68] confer or increase resistance to different β-lactams in *S. pneumoniae*. Some of them, i.e., mutations affecting CiaRH and CpoA, occur independently of *pbp* mutations. The CiaRH system is constituted of the histidine kinase CiaH and the response regulator CiaR. Recognition of an external stimulus by the surface-exposed region of CiaH triggers CiaH autophosphorylation, and the phosphoryl group is then transferred to CiaR. Phosphorylation of CiaR activates gene expression by interacting with the regulatory regions of the target genes. In streptococci, CiaRH negatively regulates competence development and expression of virulence factors, and it triggers biofilm formation [69]. CiaRH also controls the level of the lipid carrier for the transport of PG precursors. Certain mutations in CiaH enhance phosphorylation of CiaR, resulting in more lipid-linked murein precursors that counteract cell damage caused by the antibiotics. Mutations in CiaH have not been reported in β-lactam-resistant *S. suis* isolates yet, but the CiaRH system is highly conserved in streptococci [69]. CpoA is a glycosyltransferase that transfers a galactose residue to monoglucosyl-diacylglycerol, which is the lipid anchor for lipoteichoic acids [70]. Point mutations in *cpoA* might increase the production of lipoteichoic acids that could counteract the damage caused by β-lactams on the cell wall. Streptococcal PG can be heterogeneously composed of branched and unbranched subunits that generate indirect and direct cross-linking bridges in the cell wall, respectively. Penicillin-resistant *S. pneumoniae* strains contain higher levels of indirect crosslinking in the cell wall compared with penicillin-susceptible isolates [71]. Probably, alterations in PBPs that result in antibiotic resistance may alter the specificity of PBPs for branched lipid II over unbranched lipid II. MurN and MurM are part of the biosynthetic route for branched PG precursors. Both are non-essential aminoacyl-tRNA-dependent ligases that add amino acids to the lysine in the third-position of the pentapeptide stem of lipid II [72]. Particularly, MurM can attach either L-serine or L-alanine at the first position of the dipeptide bridge. MurM from penicillin-resistant *S. pneumoniae* preferentially incorporates L-alanine, while MurM from penicillin-sensitive strains preferentially incorporates L-serine. Deletion of the *murM* gene in penicillin-resistant pneumococci depletes indirect crosslinks from the PG and results in the loss of penicillin resistance [73]. Thus, MurM is necessary for high levels of resistance but not sufficient. Also, mutations within *murM* enhance antibiotic resistance [68]. This might result in new species of branched peptides with superior binding to mutated PBPs. As the *cpoA* and *murM* genes are present in *S. suis* genomes, mutations in these genes could possibly also contribute to β-lactam resistance, but, so far, they have not been reported yet.

### Resistance to the macrolide, lincosamide, and streptogramin (MLS) group

MLS antibiotics exert their antimicrobial action by binding to the 23S rRNA of the 50S subunit of the bacterial ribosome causing dissociation of peptidyl-tRNAs (Figure 2B). Consequently, MLS block translation. This group is composed of three chemically different molecules.

Macrolides consist of a macrocyclic lactone ring of 12, 14, 15, or 16 atoms (see erythromycin in Figure 3). The ring is linked to side chains, including specific sugar residues, which are varying between macrolide types. Macrolides block ribosome function by binding to the exit tunnel through which the nascent proteins leave the ribosome. The bound macrolide stalls the ribosome when it needs to polymerize specific amino-acid sequences generally called macrolide arrest motifs (MAMs) [74]. Thus, when a protein lacks MAMs, it can be synthesized in the presence of macrolides. MAM sequences vary for each macrolide type, which influences its antibiotic activity. The broader the variety of MAMs a macrolide recognizes, the higher the chance it stops translation. Also, the macrolide structure impacts the affinity and dynamics of drug-ribosome interaction, which correlates with the bacteriostatic or bactericidal activity of each macrolide.

There are three lincosamides approved for veterinary use: lincomycin, pirlimycin, and clindamycin. Lincomycin consists of a *trans*-*N*-methyl-4-*n*-L-proline linked via a peptide bond with 6-amino-6,8-dideoxy-1-thio-D-*erythro*-α-D-galactopyranoside (Figure 3). It is dedicated to use in companion animals, and it can also be applied to pigs, particularly in the case of infectious arthritis such as caused by *S. suis*. In contrast to macrolides, lincosamides directly inhibit the peptidyl transferase center of the ribosome.

Streptogramins include two different classes of molecules, called type A and type B. Type A streptogramins are polyketide-amino acid hybrids linked to the N-terminal side of an oxazole ring derived from serine. They inhibit protein synthesis by blocking substrate attachment to both the A and P sites of the ribosome (Figure 2B). Type B streptogramins are peptides cyclized through an ester bond between the carboxyl group of the C-terminal phenylglycine and the hydroxyl group of a threonine residue. They inhibit peptide bond formation during elongation by causing incorrect positioning of the peptidyl tRNA at the P site of the ribosome, particularly upon incorporation of proline or basic amino acids.

Resistance to MLS antibiotics is generated by various mechanisms. One of the most widespread mechanisms is methylation of the ribosome at the macrolide-binding site, i.e., the adenine at position 2058 (A2058) located in the variable region of the 23S rRNA in *E. coli*, by erythromycin-resistance methyltransferases encoded by *erm* genes. Erm enzymes add one or two methyl groups to the N6 amino group of A2058, thereby preventing the formation of a key hydrogen bond between A2058 and the desosamine sugar at C5 of the macrolide. The acquisition of MLS resistance by ribosome methylation reduces bacterial fitness [75]. However, methylation can be regulated translationally or transcriptionally. About 40 *erm* genes have been reported, which are grouped in 14 *erm* classes. The classes found in *S. suis* are listed in Table 1. The *erm*(B) class is the most common macrolide-resistance determinant in *S. suis* [76], while the *erm*(G) [77] and *erm*(T) classes [78] are less frequently found [62]. Indeed, ribosome methylation by Erm(B) confers a high level of resistance not only to macrolides but also to lincosamides and streptogramin B. This phenotype is called a macrolide-lincosamide-streptogramin B resistance phenotype (often referred to as MLS_(B)_). In contrast to other methylases that require the presence of specific drugs for induction of their synthesis, the synthesis of this methylase is constitutive, which leads to the development of resistance to all MLS_(B)_ drugs. Cfr is a methyltransferase that methylates nucleotide A2503 of the 23S rRNA causing combined resistance to MLS, amphenicols, and pleuromutilins (known as PhLOPS_A_ phenotype) [79]. The A2503 residue is present in the overlapping binding sites of the mentioned antibiotics, and its methylation interferes with the position and binding of the drugs. Thus, Cfr prevents the binding of antibiotics to the ribosome. The *cfr* gene has been found in various Gram-positive species, such as *Staphylococcus aureus* [80], *Bacillus* spp. [81], and *Enterococcus* spp. [82], where it is located on plasmids or on the chromosome. The *cfr* gene was first identified in a florfenicol-resistant *S. suis* isolate from a healthy pig in China in a routine surveillance study [83]. In *S. suis* strain 10, it was found to be present on a 100-kb plasmid, where it was flanked by two copies of the insertion sequence IS*Enfa5* [84], probably contributing to its dissemination.

Drug-inactivating enzymes can also mediate resistance to various MLS antibiotics. Examples are the phosphorylation of the 2′-hydroxyl group of the amino sugar and hydrolysis of the macrocyclic lactone exerted by phosphotransferases and esterases, respectively, or the chemical modification of lincosamide with either phosphate or adenylate groups. Lincosamide nucleotidyltransferases inactivate only lincosamides. They comprise members of the *lnu* (previously *lin*) gene family, of which different types were identified, i.e., *lnu*(A), *lnu*(B), *lnu*(C), *lnu*(D), *lnu*(E), and *lnu*(F), most of which have also been identified in *S. suis* isolates (Table 1).

Besides their modification, also their active export can confer resistance to various MLS antibiotics in *S. suis* (Table 1). However, in contrast to resistance mediated by target-modifying enzymes, the resistance conferred by specific pumps is often specific for each antibiotic type. An example is the Mef(E)/Mel system that confers resistance to macrolides. Mef(E) is a protein of 405 amino-acid residues that belongs to the major facilitator superfamily (MFS) and that expels macrolides from cells by using the proton-motive force as the energy source. Mel, a.k.a. Msr(D), is a homolog of ATP-binding cassette (ABC) transporter proteins but misses membrane-spanning domains [85]. These proteins are called ABC-F proteins and contain two ATP-binding cassettes separated by a linker of about 80 amino-acid residues. Presumably, Mel displaces ribosome-bound macrolides after which it may transfer them to Mef(E) for efflux [86]. As such, Mef(E) and Mel operate as a two-component efflux pump [85, 87]. Several Mef(E) variants have been described, i.e. Mef(A) and Mef(I). They share around 90% of sequence identity with Mef(E) and, therefore, they are not always distinguished in the literature. In *S. suis*, *mef*(A) and/or *mef*(E) have been identified [88]. Lincosamide resistance can be conferred by the ABC-F proteins of the Vga and Lsa families, but, unlike in the case of Mel, there are no analogues of Mef(E) protein involved. Thus, Vga and Lsa could primarily function as ribosome-protection proteins (RPP). Genes encoding the ABC-F proteins Lsa(E) and Vga(F), which confer resistance to lincosamides, streptogramins A, and pleuromutilins, have been detected in *S. suis* [89]. Finally, mutations affecting ribosomal components could potentially also confer MLS_(B)_ resistance in *S. suis*. Substitutions of A2058, A2059, and C2611 in the 23S rRNA (nucleotide numbering according to *E. coli*) [90] and mutations in ribosomal proteins L4 and L22 generate macrolide resistance. L4 and L22 are ribosomal proteins with domains on the surface of the ribosome and at the exit tunnel near the macrolide-binding site [91]. Such substitutions in the 23 rRNA and in the L4 and L22 proteins have been described in *S. pneumoniae* [92], but not yet in *S. suis*.

### Resistance to amphenicols and pleuromutilins

Like MLS antibiotics, amphenicols and pleuromutilins bind to the 23S rRNA of the 50S subunit of the bacterial ribosome, thereby blocking translation (Figure 2B). They are chemically different from MLS (see examples in Figure 3) but, nevertheless, they share overlapping binding sites.

Amphenicols comprise a series of molecules with a monocyclic core. The first molecule of this class was chloramphenicol isolated from *Streptomyces venezuelae*. However, chloramphenicol produced serious side effects and, therefore, it was replaced by synthetic analogues. Thiamphenicol is a chloramphenicol derivative that contains a methylsulfonyl group instead of a *p*-nitro group. Florfenicol is a fluorinated derivative of thiamphenicol with a fluorine group replacing a hydroxyl group at C3 (Figure 3). Florfenicol is approved for use in food production animals and has been used to treat *S. suis* infections. Pleuromutilins contain a diterpene structure. The first antimicrobial pleuromutilin was isolated from *Pleurotus mutilus* [93]. Later, synthetic analogues were produced, including tiamulin and valnemulin, which were approved exclusively for veterinary use in food production animals [94]. Tiamulin (Figure 3) is broadly used to treat *S. suis*.

As for MLS antibiotics, amphenicol and pleuromutilin resistance can be acquired by several routes, including target-site modification, enzymatic inactivation of the antibiotics, and active efflux (Table 1). Some of these mechanisms are common to amphenicols, MLS, and/or pleuromutilins, because of their similar mechanism of action (Table 1). For example, the substitution of guanine at position 2032 in the 23S rRNA confers resistance to chloramphenicol, clindamycin, and pleuromutilins, and the substitution of guanine at position 2576 confers resistance to chloramphenicol and clindamycin (reviewed in Schwarz et al. [95]).

Production of chloramphenicol O-acetyltransferases (Cats) inactivates amphenicols by transferring an acetyl group from acetyl-S-coenzyme A to the C3 or C1 positions of the amphenicol molecule generating mono- or di-acetylated derivatives, which lack antimicrobial activity [96]. However, they can not inactivate florfenicol, due to the presence of a fluorine instead of a hydroxyl group at the C3 position (Figure 3). There are two types of Cats, called CatA and CatB. CatA can be further classified into 22 different groups based on their percentage of indentity; they are broadly distributed in Gram-positive and Gram-negative bacteria. CatB can be classified in five subtypes; they are related to acetyltransferases involved in streptogramin A resistance. As compared to CatA enzymes, CatB confers lower MICs. CatA-encoding genes have been described in *S. suis* [62, 97], but their prevalence is rather low. This could be explained by the inactivity of these enzymes against florfenicol or by the prohibition of the use of chloramphenicol for food-production animals because of toxicity issues. This *catA* gene and an upstream-located *optrA* gene*,* another amphenicol-resistance determinant, are flanked by two IS*1216* elements, allowing their co-mobilization [97]. Pleuromutilin-inactivating enzymes have not been described so far.

Amphenicols and pleuromutilins can be expelled from the bacterial cells using broad-spectrum exporters or specific transporters. A specific amphenicol exporter described in Gram-positive bacteria is FexA. FexA is an MFS exporter. Its expression is inducible with chloramphenicol or florfenicol, and it confers resistance to both antibiotics. The *fexA* gene was first found in a florfenicol-resistant *S. suis* isolate, located on a ∼100-kb plasmid, designated pStrcfr, together with a *cfr* gene [84]. However, its distribution and activity are controversial. In most of the studies analyzing AMR determinants in *S. suis*, *fexA* is not reported. For example, in a recent screening, 14% of 148 Australian *S. suis* isolates showed florfenicol resistance, but only one carried a *fexA* gene. In contrast, in a recent study in Spain, a *fexA* gene was found in 25% of the tested isolates but, curiously, it was not related to florfenicol resistance [8]. A possible explanation is that the detected *fexA* is a non-functional variant. In fact, a *fex*A variant that confers resistance only to chloramphenicol was detected in a canine *S. pseudintermedius* isolate [98]. This is caused by two mutations, Gly33Ala and Ala37Val, both of which are critical for substrate recognition and, therefore, the encoded transporter is inactive for florfenicol. This new variant is not distinguished by PCR from the wild type, which could explain the lack of association between the presence of *fexA* and florfenicol resistance in the Spanish isolates. However, considering that chloramphenicol is prohibited for use in Spain since more than a decade ago, this new *fexA* variant, which appears to be widespread within recent *S. suis* isolates, might be associated with the export of other AMR determinants. Thus, the role of *fexA* in amphenicol resistance in *S. suis* remains unclear. Also, the ABC-F RPP variants Lsa(E) and Vga(F) confer resistance to pleuromutilins [99], and both have been detected in *S. suis* [89].

In recent years, *optrA* has emerged as a determinant of resistance to amphenicols and oxazolidinones, a class of antibiotics that inhibit translation by binding the ribomome P site. Initial studies characterized OptrA as an ABC transporter [100], but later work showed that it is an RPP protein of the ABC-F family [86]. The *optrA* gene was frequently detected in *S. suis* isolates from China with a prevalence ranging from 11% [33, 97] to 38% [83]. An initial genetic analysis of publicly accessible *S. suis* genome sequences revealed that *optrA* was flanked downstream by an IS*1216E* element in five out of six genomes. In one genome, it was located together with several resistance genes on a genetic segment flanked on either side by IS*1216E*, and in two genomes, it was located within pathogenicity islands and conjugative elements (described in Sect. 4). In the remaining genomes, the *optrA* gene was integrated in a large prophage genome [101]. A later study also located an *optrA* gene on a 40-kb plasmid [33]. Hence, these analyses suggest that *optrA* was acquired by horizontal gene transfer (HGT), probably from *Enterococcus* [100], and spread further among *S. suis* via mobile genetic elements (MGE, described in next section). A *cfr* gene (discussed above) also confers resistance to amphenicols and pleuromutilins and, as *optrA*, it was found in MGEs [84]. Its prevalence is rather low (< 1%) as compared with *optrA* (38%) [83], suggesting that *optrA* is a more relevant determinant for AMR in *S. suis*.

### Resistance to tetracyclines

Tetracyclines comprise a family of broad-spectrum antibiotics with activity against Gram-positive and Gram-negative bacteria. Their structures share four rings linked to various substituents including amine, chloride, or hydroxyl groups (see doxycycline in Figure 3). Tetracyclines bind to the 16S rRNA in the 30S ribosomal subunit (Figure 2B), arresting translation by sterically interfering with the docking of aminoacyl-tRNA during elongation [102]. Thus, as MLS, amphenicols, and pleuromutilins, tetracyclines also inhibit translation, but their binding site is different. Uptake of tetracycline into the cytoplasm can be mediated by passive diffusion or active transport.

Resistance to tetracyclines can be attributed to different mechanisms, including (i) active drug export, (ii) ribosome protection, and (iii) drug inactivation. Target-site modifications have, so far, only been reported in other bacteria. For example, the substitutions C1054T and T1062G/A in the 16S rRNA conferred resistance to tigecycline in *S. pneumoniae*, and the resistance level was incremental with the number of the four genomic copies for the 16S rRNA that acquired the mutations [103]. Besides, mutations in genes for ribosomal proteins can result in tetracycline resistance. For example, mutations in the *rpsJ* gene resulting in substitutions or deletions within residues 53–60 of the 30S ribosomal subunit protein S10 conferred tetracycline and tigecycline resistance. Also, mutations in the *rpsC* gene resulting in Lys4Arg and His157Asp substitutions in ribosomal protein S3 were associated with reduced tigecycline susceptibility in *S. pneumoniae* [103].

Apart from target-site mutations, protection to tetracyclines can be generated by RPPs of the ABC-F family. These are GTPases that release the bound tetracycline from the ribosome. RPPs have structural similarity to elongation factors EF-G and EF-Tu. Conformational changes induced by RPPs promote the formation of the EF-Tu-GTP-aminoacyl-tRNA ternary complex, which allows translation to proceed in the presence of tetracycline [104]. There are currently 12 reported RPP genes; some of them can protect against multiple drugs, such as tetracycline, minocycline, and doxycycline, while others are more specific and do not inhibit the activity of some tetracyclines, such as tigecycline and other glycylcyclines. The best characterized RPPs are Tet(O) and Tet(M), which share about 75% of sequence similarity. The corresponding genes are frequently identified in tetracycline-resistant *S. suis* isolates worldwide [8, 39], and new variants were recently identified [62]. Interestingly, autoinducer 2 (AI-2), which stimulates gene expression for biofilm formation and increases growth rates, upregulates the expression of Tet(M) in *S. suis* [105]. Other RPPs are those encoded by *tet*(S), *tet*(44) and *tet*(W), which were identified in *S. suis* isolates from Asia [51], America [62] and United kingdom [62]*.*

The most common tetracycline-specific efflux pumps are MFS transporters [106]. These pumps extrude tetracyclines from the cells at the expense of the proton-motive force and are classified in seven different groups based on amino-acid sequence similarities and the number of transmembrane segments [107]. Clinically, the most prevalent pumps are members of either group 1 or group 2. The group 2 pumps are present in Gram-positive bacteria and include Tet(K) and Tet(L); both of them, together with *tet*(B), and *tet*(40), were identified in *S. suis* (Table 1)*.*

### Resistance to sulfonamides and trimethoprim

Sulfonamides are polar molecules which contain a sulfonyl group connected to an amine group. More than 5000 derivatives have been developed, of which sulfathiazole (Figure 3), sulfamethazine, and sulfadiazine are the main ones used in veterinary medicine. Sulfonamides act as inhibitors of the enzyme dihydropteroate synthase. This enzyme catalyzes the conversion of *p*-aminobenzoic acid and 7,8-dihydro-6-hydroxymethylpterin-pyrophosphate into dihydropteroate, a precursor in the folate synthesis pathway (Figure 2C). Folates are important cofactors required to produce amino acids and nucleotides. Thus, as this is an essential process for growing bacteria, sulfonamides have a broad spectrum of activity against Gram-positive and Gram-negative microorganisms.

To the best of our knowledge, the mechanisms of resistance to sulfonamides have not been described so far in *S. suis*. In other pathogenic streptococci, the main mechanism of resistance correlates with mutations in conserved regions of *folP*, the gene that codes for dihydropteroate synthase. Specific alterations in the amino-acid sequence of the enzyme result in the loss of affinity for sulfonamides [108]. These mutations seem to occur spontaneously at various positions within the gene. For example, spontaneous sulfonamide-resistant *S. pneumoniae* mutants obtained in laboratory contained a 6-nucleotide duplication resulting in alteration of the tertiary structure of the enzyme [109], whereas several clinical resistant isolates also contained oligonucleotide duplications at different positions [110] or single nucleotide substitutions resulting in Ile100Leu or Glu20Asp substitutions in the enzyme [111]. Diverse studies on clinical sulfonamide-resistant *Streptococcus pyogenes* and *Streptococcus mutans* isolates reported a variety of mutations in the *folP* gene [112]. An alternative mechanism is tandem gene duplication. Studies in *Streptococcus agalactiae* isolates revealed a fourfold tandem amplification of a chromosomal DNA fragment carrying all five genes required for dihydrofolate biosynthesis including *folP*, which led to sulfonamide resistance [113]. Interestingly, trimethoprim, is a competitive inhibitor of dihydrofolate reductase (encoded by *dhfr* gene) that is a part of the folate production pathway (Figure 2). However, trimethoprim is a 2, 4-diamino-5–3´-trimethoxybenzyl pyrimidine (Figure 3) that belongs to diaminopyrimidines group, therefore is not a sulfonamide drug. It is often co-administrated with sulfamethoxazole because of the synergic activity of both antibiotics on the same pathway. Mutations in *S. suis dhfr* and its promoter were associated with reduced susceptibility to trimethroprim, as well as horizontal acquisition of transmissible trimethoprim-insensitive *dhfr* genes [62] (Table 1), as found in other streptococcus species.

### Resistance to aminoglycosides

Aminoglycosides are antibiotics composed of a core structure of amino sugars linked to a dibasic aminocyclitol by a glycosidic bond (Figure 3). They are polycationic structures that bind to the negatively charged components of the bacterial membrane such as teichoic acids and phospholipids or, in Gram-negative bacteria, lipopolysaccharides. This activity causes magnesium displacement, a process that enhances membrane permeability and facilitates antibiotic entry. Some aminoglycosides are still active against *Streptococcus* and *Enterococcus* spp., e.g*.* tobramycin (Figure 3), but only at higher concentrations as compared to other bacteria because of natural resistance. They inhibit protein synthesis by binding to the A-site on the 16S rRNA (Figure 2B). Aminoglycoside-ribosome interaction causes codon misreading, resulting in incorrect assembling of amino acids. Different aminoglycosides have different ribosome specificities.

Aminoglycoside resistance is caused by different mechanisms, including enzymatic modification of the antibiotic, target-site modification via an enzyme, and efflux. Aminoglycoside-resistance mutations altering the ribosome have not been reported in *S. suis*. Aminoglycoside-modifying enzymes are widespread. Aminoglycoside modifications include acetylation, phosphorylation, and adenylation at different positions in the aminoglycoside [114]. Modification decreases the affinity of the drug for its target. Aminoglycoside-modifying enzymes comprise three families, i.e., aminoglycoside N-acetyltransferases (AACs), aminoglycoside O-nucleotidyltransferases (ANTs), and aminoglycoside O-phosphotransferases (APHs). The families are further divided into subtypes according to the position on the aminoglycoside that is modified. ANTs transfer AMP from ATP to the hydroxyl groups at positions 2″, 3″, 4’, 6’, or 9’ of the aminoglycoside. Several genes coding for ANTs have been discovered in aminoglycoside-resistant *S. suis* isolates. Examples include *ant1* [62], *ant*(6´)-Ia [115, 116], *ant*(6´)-Ib [62], and *ant*(9´)-Ia [116] (Table 1). AACs comprise a large group of enzymes that acetylate amino groups at different positions on the aminoglycoside. There are four subclasses of AACs based on the position of the amino groups that are modified. The *aac*(6´) gene was identified in various multidrug-resistant *S. suis* isolates from Asia with high MIC values for aminoglycosides [115, 117]. It is often fused to other genes coding for different aminoglycoside-modifying enzymes, for example APHs (Table 1), thus generating bifunctional enzymes. APHs catalyze the ATP-dependent phosphorylation of hydroxyl groups on aminoglycosides [118]. In *S. suis*, several genes only coding for an APH variant, such as *aph*(3´)-IIIa and *aph(6)-Ia*, have been reported [88, 119] (Table 1).

### Resistance to glycopeptides

Glycopeptides are a group of glycosylated cyclic or polycyclic peptides (Figure 3). They act by binding the D-alanyl-D-alanine terminus of cell wall precursors (Figure 2A), thus preventing their incorporation into the PG strands. Representative examples are vancomycin, teicoplanin, telavancin, dalbavancin, and oritavancin. These antibiotics are exclusively used in humans, but ovoparcin (Figure 3) has been used as growth promoter in pig production. Structural differences in these antibiotics have implications for their mechanism of action. For example, vancomycin has higher affinity for the PG precursors than teicoplanin, which is based on dimer formation. In contrast, teicoplanin interacts with the lipid bilayer of the bacterial membrane resulting in its localization near the lipid II substrate. However, these antibiotics can also select for resistance traits that could be transferred to human pathogens via food chain.

Glycopeptides are large molecules that cannot cross the outer membrane in Gram-negative bacteria through porins and, therefore, Gram-negative bacteria are intrinsically resistant to glycopeptides. In contrast, Gram-positive bacteria are susceptible. The main glycopeptide-resistance mechanism developed by Gram-positive bacteria is the production of substrate-modifying enzymes that reduce the affinity of the substrates for the antibiotics. By their action, the carboxy-terminal D-alanine residue of PG precursors is replaced by either D-lactate or D-serine [120]. There are several classes of cell-wall modification systems that confer resistance to glycopeptides. They are encoded by gene clusters referred to as *van*. The *vanA*, *vanB*, *vanD*, *vanM*, and *vanF* clusters code for enzymes that replace the terminal D-Ala by D-lactate, whereas the clusters *vanC*, *vanE*, *vanG*, and *vanL* code for enzymes that replace the terminal D-Ala by D-Ser. Several *van* genes were identified in *S. suis* (Table 1), including the *vanG* operon [121] and *vanZ* gene [122]. The presence of a *vanG* operon results in an intermediate level of resistance to vancomycin [123]. The *vanG* operon is composed of several genes: (i) *vanG*, which encodes a D-Ala-D-Ser ligase, (ii) *vanX* and *vanY*, which encode a putative D,D-peptidase and D,D-carboxypeptidase, respectively, (iii) *vanT*, which codes for a serine racemase, and (iv) *vanR* and *vanS*, whose products constitute a two-component regulatory system that controls expression the operon. The *vanZ* gene is usually located within the *vanA* gene cluster, but this is not the case in *S. suis* and *Clostridioides difficile* (previously called *Clostridium difficile*) [124]. The function of the VanZ protein remains unknown, but it increases teicoplanin resistance in *Enterococcus faecium* and *C. difficile*, and has no impact on vancomycin resistance [124, 125].

### Resistance to quinolones

Quinolones are molecules composed of a basic bicyclic core, which may contain a fluorine atom (fluoroquinolones), usually at the C6 position, and various other substitutions (see enrofloxacin and norfloxacin in Figure 3). Depending on the core structure, they can be classified into four groups: (i) monocyclic, (ii) bicyclic, (iii) tricyclic, and (iv) tetracyclic derivatives, and, based on the position of the fluorine atom, each group can be subdivided into subgroups. Quinolones are derivatives of the synthetic nalidixic acid, first reported in 1962. Since then, nalidixic acid analogues were elaborated and optimized over time [126]. Examples of the second generation are norfloxacin (Figure 3) and ciprofloxacin. The third and fourth generations encompass fluoroquinolones with broader activity, efficacy, and lower resistance development than previous analogues. Relevant antibiotics are levofloxacin (third generation) and moxifloxacin (fourth generation), which are more active against Gram-positive bacteria than their predecessors [127]. Of the different quinolones generated, ciprofloxacin, norfloxacin, and enrofloxacin (Figure 3) have been and are broadly used to treat *S. suis* infections.

Quinolones target bacterial type II topoisomerases (Figure 2D), i.e., DNA gyrase and topoisomerase IV. These enzymes have crucial functions by catalyzing the interconversion of topological forms of DNA [128]. Although their working mechanism is similar, their biological roles are related but not identical. In contrast to topoisomerase IV, DNA gyrase generates negative supercoils into DNA, and it removes the torsional stress generated during replication and transcription from the DNA. Also, topoisomerase IV removes torsional stress, but, in addition, it mediates the separation of the two daughter chromosomes (decatenation) after DNA replication [129]. Both enzymes are composed of two subunits forming hetero-tetrameric complexes, i.e., GyrA and GyrB in the case of gyrase and ParA and ParC in the case of topoisomerase IV. For their activity, both enzymes have ATP-dependent DNA cleaving and ligating activity [128]. Quinolones intercalate between DNA bases at the DNA cleavage-ligation site, thereby inhibiting ligation. Thus, bacterial exposition to quinolones increases the concentration of cleaved DNA leading to cell death. Quinolones have different preferences for their targets. In Gram-negative bacteria, quinolones have higher affinity for DNA gyrase than for topoisomerase IV. However, in some Gram-positive bacteria, including *S. pneumoniae*, topoisomerase IV rather than gyrase is the primary target. Furthermore, in *S. pneumoniae*, the quinolones nature also determines the affinity for the target [130].

Quinolone resistance is grouped in several categories, including (i) alteration of quinolone targets, (ii) production of antibiotic-modifying enzymes, and (iii) enhanced production of efflux pumps. In *Streptococcus* species, including *S. suis*, two main mechanisms have been described: mutations at the target site and enhanced production of efflux pumps (Table 1). Quinolone resistance is often associated with chromosomal mutations in the gyrase- and/or topoisomerase IV-encoding genes. Combinations of mutations in both enzymes yield high levels of resistance. In *S. suis*, mutations in *gyrA, gyrB, parC*, and *parA* have been described and related to quinolone resistance (Table 1) [62, 88, 131]. The most frequent mutations occur in *gyrA* at position Ser81 and, less frequently, at position Glu85. Also, structural analysis identified amino acids involved in the binding of a quinolone via a Mg^2+^ ion by forming hydrogen bonds to water molecules that coordinate the Mg^2+^ ion. Thus, their substitution interferes with quinolone binding [132]. Curiously, these amino-acid residues are highly conserved within the bacterial kingdom, although their involvement in protein function remains unclear. To a lesser extent, quinolone-resistance mutations are found also at positions Ser79 and Asp83 in ParC of *S. suis* [62, 88]. This is in contrast to other Gram-positive bacteria, where mutations in *parC* are first to occur [133]. These data suggest that DNA gyrase is the main target for the quinolones used to treat *S. suis*. However, several isolates have mutations in both genes [88], probably as a mechanism to increase resistence levels.

Another mechanism of resistance to fluoroquinolones in Gram-positive bacteria is the increased production of efflux pumps. In Gram-positive bacteria, several members of the MFS, the multiple antibiotic- and toxin-extrusion (MATE), and the ABC-transporter families recognize quinolones as substrates [134]. In *S. suis*, the ABC transporter SatAB has been reported to be involved in this function [135]. SatAB exports norfloxacin and ciprofloxacin [131, 135]. On the chromosome, the *satA* and *satB* genes are organized in an operon. The regulation of *satAB* expression is complex and controlled by different molecules. SatR, a MarR-family regulator, acts as a repressor of the operon [136]. Besides, the operon is regulated by the quorum-sensing system LuxS/AI-2 in *S. suis* [137]. Particularly, AI-2 upregulates the expression of the *sat* genes, thus increasing efflux pump production, leading to increased quinolone resistance [137]. SatAB is homologous to pneumococcal PatAB, an ABC transporter that provides resistance to norfloxacin, ciprofloxacin, and levofloxacin [138]. The expression of PatAB is upregulated by exposition of the bacteria to quinolones [139]. Thus, it is expected that SatAB of *S. suis* is also regulated by quinolones, but this hypothesis requires experimental evidence. Besides, the gene with locus tag SS2069 was found to be upregulated in quinolone-resistant *S. suis* isolates carrying the anticipated mutations in the type II topoisomerases [131]. SS2069 codes for an extracellular protein that is part of an ABC transporter. This extracellular location is difficult to conciliate with a direct role in the export of quinolones out of the cells and, thus, its role in quinolone resistance remains enigmatic. In pneumococci, quinolone export is also driven by PmrA [140], an MFS-type efflux pump that exports a variety of substrates including norfloxacin, ethidium bromide, and acriflavine. A *pmrA* gene is also present in *S. suis* genomes (i.e., SSU1222 in P1/7 genome), but its contribution to quinolone resistance remains to be demonstrated.

The production of enzymes that alter the antibiotic targets or the quinolones has been identified in other bacteria, particularly in Gram-negative bacteria. Interestingly, a variant of the aminoglycoside acetyltransferase AAC(6´)-Ib generating a moderate resistance to ciprofloxacin has been identified in Gram-negative bacteria [141]. This enzyme inactivates ciprofloxacin by N-acetylation. This is not surprising, as AAC belongs to a superfamily of enzymes that modify a large variety of substrates. Remarkably, *aac*(6´) variants have been identified in various multidrug-resistant *S. suis* isolates from Asia with high MIC values for aminoglycosides [115, 117] (Table 1). In Gram-negative bacteria, the *aac*(6´)-Ib gene is transferred by plasmids, but this is not a usual mechanism in Gram-positive bacteria. However, the wide distribution of *aac* genes in *S. suis* genomes leads us to speculate that these enzymes could undergo adaptation to modify other antibiotics such as quinolones.

## Transfer of antibiotic-resistance genes

*S. suis* is considered a reservoir of AMR genes, which are shared among *S. suis* clones and can also be transferred to other bacterial species by HGT [142, 143]. HGT can be mediated by MGEs during conjugation process or simply by DNA fragments by transformation process. MGEs are DNA elements that can be transferred between cells and/or within a genome. Many MGEs harbor genes for mobility, i.e., integration, excision, and conjugation. Genes involved in separate functions of the mobilization process are often clustered together and, thus, MGEs show a modular organization. In addition, MGEs can carry a diversity of genes for metabolic pathways, virulence factors, symbiosis, interbacterial competition, and/or AMR. Such genes confer an advantage to the host in a particular niche, which favors the selection of clones that acquired the MGE [144]. Clearly, *S. suis* prefers to share its AMR genes via MGEs. This was illustrated in a recent study which revealed that all AMR genes identified in 214 genomes of drug-resistant *S. suis* isolates of 26 different serotypes were located on MGEs [145].

Most MGEs use well-described mechanisms for their mobilization, such as type I and type II transposons, insertion sequences, plasmids, prophages, and chromosomal integrative elements transferring by conjugation. The latter elements include (i) integrative and conjugative elements (ICEs), which are autonomous in transfer and integration, (ii) integrative and mobilizable elements (IMEs), which are autonomous for excision and integration but not for transfer, (iii) elements that are autonomous for transfer but not for integration (i.e., deviating from an ICE). More recently, unconventional MGEs have been discovered, but the mechanisms for their mobility have not been identified yet. Regardless of the presence of genes related to mobilization and integration, MGEs contain genetic features, such as an unusual G + C content or codon usage, that indicate that they were acquired by HGT. Anyway, MGEs can be exchanged between bacteria by different mechanisms, including conjugation, transformation and transduction [146]. Other mechanisms implicated in HGT involve the release of membrane vesicles or elongated membranous structures named nanotubes.

### Conjugation

Conjugation is the most frequently used mechanism of AMR-gene transfer in *S. suis*. Two categories of chromosomal elements can be transferred by conjugation, i.e., ICEs and IMEs [147]. ICEs contain all genetic information needed to mediate their autonomous excision, conjugation, and chromosome integration [147]. They are also known as conjugative transposons. They can also carry genes that mediate heavy-metal resistance, AMR, and/or biofilm formation, amongst others [146]. ICEs are excised from the chromosome by site-specific recombination at the *att* sites (*attL* and *attR*), a process mediated by tyrosine and serine recombinases or DDE transposases. After excision from the chromosome, ICEs are circularized and then transferred to a recipient cell by conjugation. To achieve this, the donor and the recipient must establish intimate contact, which is mediated by pili and/or adhesins at the cell surface. Then, the DNA is transferred by a conjugation apparatus that comprises a relaxase, called MOB, a mating-pair formation system, which is constituted by a membrane-spanning multi-protein complex, known as type IV secretion system (T4SS), and a coupling protein located at the inner side of the membrane [144]. The relaxase binds to the origin of transfer, *oriT*, on the ICE, cleaves one strand, and forms a covalent bond with the 5´ end of the cleaved strand. The coupling protein binds the DNA bound-relaxase to the T4SS. Rolling-circle replication displaces the cleaved strand, and the relaxase and the single strand are actively transferred from the donor through the T4SS to the recipient, where the complementary strand is synthesized. Finally, ICEs can be integrated into the recipient chromosome at various sites, often located in tRNA genes but also in various house-keeping genes [144]. IMEs undergo autonomous excision and integration but, in contrast to ICEs, they are not equipped with a full set of genes for conjugation. Even so, IMEs can contain genes for a relaxase or even a coupling protein and other conjugation-related gene can be present. Nevertheless, they parasitize on conjugative elements for mobility. In fact, many IMEs are integrated into ICEs. Even so, these elements correspond to different categories of MGEs since their transfer functions are genetically unrelated or only distantly related.

ICEs frequently mediate the transfer of AMR genes in *S. suis* and are responsible for multidrug-resistant phenotypes. Table 2 lists the characteristics of some representative ICEs found in *S. suis* and harboring AMR genes (reviewed in Dechêne-Tempier et al. [53]). They are broadly distributed among *S. suis* genomes. In silico analysis of 214 *S. suis* genomes revealed the presence of 242 complete ICEs and 135 derivative ICEs, i.e., ICEs containing truncated genes for mobilization [145]. The families of ICEs described in *S. suis* genomes are Tn*916* [148], Tn*5252* [148], Tn*1549* [145], Tn*GBS2* [145], Tn*GBS1* [145], ICE*St3* [145], and *vanG* [145]. Most of them encode a canonical relaxase of the MOBp family associated with a coupling protein of the VirD4 family. *S. suis* ICEs are mostly integrated in house-keeping genes, including *rplL*, *rumA*, *mutT*, a luciferase-like monooxygenase gene (SSU0468, *llmO*), and *rbgA*, among others, or in non-coding sequences, as well as in other MGEs (see Table 2). The integration sites vary between ICE families, and there may be more than one insertion site for each family (Table 2). AMR genes are present in several *S. suis* ICE families, but they are most frequently found in ICEs of the Tn*5252* family [145]. AMR genes have so far not been detected in ICE families Tn*GBS1* and ICES*t3* carried by *S. suis*. A large variety and different combinations of AMR genes can be found in a single ICE (Table 2). It has been demonstrated that ICEs can transfer AMR genes between *S. suis* strains and also to other streptococci, such as *S. pneumoniae* and *S. agalactiae* [148], and even to bacteria of other genera. In fact, many ICEs are found in several different bacterial species (Table 2). An example is ICE*Ss*D9, which carries the erythromycin- and tetracycline-resistance determinants *erm*(B) and *tet*(O) (Table 2), respectively, and which was transferred between *S. suis* and *Enterococcus faecalis* [149]. Also, ICE*Ssu*32457 (Table 2) of *S. suis* strain 32,457 was transferable to *S. agalactiae* strain RF12 in vitro. Importantly, ICE*Ssu*32457 recombined with *S. agalactiae* ICE*Sa*2603 generating a hybrid island that was transferable to *S. pyogenes* strains [150]. Obviously, the formation of ICE hybrids can facilitate the accumulation of AMR genes and the generation of multidrug-resistant strains. Interestingly, ICE*Ssu*SC216 of *S. suis* strain SC216 and a tandem ICE designated tandem ICE*Ssu*SC317 of strain SC317, which is composed of two different consecutive ICEs, both contain an *optrA* gene (oxazolidinone/amphenicol resistance) flanked by two IS*1216* elements [97]. Both ICEs belong to Tn5252 family, but they are inserted at different locations. The *optrA* gene is also located on the prophage ΦSC181 (Table 2) in *S. suis* strain SC181 associated with a *cat* gene (chloramphenicol resistance) and an *araC*-like transcriptional regulatory gene [97], and flanked by two IS*1216* elements. Inverse PCR assays revealed that IS*1216* elements can recombine and form circular intermediates. Additional examples of ICEs carrying AMR genes in *S. suis* are listed in Table 2. Together, these observations demonstrate that AMR genes can move between various MGEs of a single strain and that independent ICEs can recombine to form tandems or hybrids. Both phenomena could explain, in part, the mosaic of AMR genes found in ICEs (Table 2), but, also importantly, they can facilitate the dissemination of many AMR genes between strains, thereby stimulating the emergence of multidrug-resistant *S. suis* strains. Supporting this notion are studies in *S. suis* strains carrying ICE*SsuBSB6*, which is composed of two regions, ARGR1 and ARGR2, both containing AMR genes. The ARGR1 region carries six resistance determinants conferring resistance to macrolides, aminoglycosides, and tetracyclines (Table 2) and shows high similarity to the island ICE*Ssu32457* and to *E. faecalis* plasmid pEF418. The ARGR2 region only possesses the glycopeptide-resistance operon *vanG*, and it is similar to the *vanG1* island of *E. faecalis* BM4518 and the *vanG2* island of *S. agalactiae* GBS-NM [121].

**Table 2:** **Examples of MGEs carrying AMR genes found in** ***S. suis*** **genomes**

MGE	Hosts	AMR genes	Integration site (s)	Integrase family	References
ICEs					
Tn*916*					
Tn*916*	*S. suis*, *S. agalactiae, S. pneumoniae, S. mutants, E. faecalis, C. difficile*	*tet*(M)	*llmO*	Tyrosine	[145]
dICE_Tn*916*	*S. suis*	*tet*(O)			[145]
dICE_Tn*916*	*S. suis*	*tet*(M)			[145]
Tn*5252*					
ICE_Tn*5252*_rplL^*1^	*S. suis, S. agalactiae, S. pneumoniae, S. pyogenes*	*tet*(O), *tet*(O/W/32/O), *tet*(40), *erm*(B), *aph*(3’)-IIIa, *ant*(9’), *ant*(6’)-Ia, *sat4*	*rplL*	Tyrosine	[89, 145]
ICE_Tn*5252*_rumA^*1^	*S. suis, S. agalactiae, S. pneumoniae, S. pyogenes*	*tet*(M), *tet*(L), *tet*(O), *tet*(O/W/32/O), *erm*(B), *aph*(3’)-III, *ant*(6’)-Ia*, sat4*	*rumA*	Tyrosine	[89, 97, 143, 145]
ICE_Tn*5252*_mutT^*1^	*S. suis*	*tet*(L), *tet*(O), *tet*(O/W/32/O), *erm*(B), *msr*(C), *mef*(A)	*mutT*	Tyrosine	[89, 145]
ICE_Tn*5252*_rbgA^*1^	*S. suis*	*erm*(B)	*rbgA*	Tyrosine	[145]
ICE_Tn*5252*_ *llmO* ^*1^	*S. suis*	*tet*(M)	*llmO*	Tyrosine	[145]
dICE_Tn*5252*	*S. suis*	*tet*(O), *tet*(40)	*rplL*	Tyrosine	[145]
Partial_Tn*5252*	*S. suis*	*tet*(O/W/32/O), *erm*(B), *aph*(3’)-IIIa, *ant*(9’), *sat4*	*rplL*	Tyrosine	[145]
ICE*Ss*D9^*2^*^3^	*S. suis, E. faecalis*	*tet*(O), *erm*(B)	*rplL*	Tyrosine	[143, 149]
ICE*Ssu*32457^*2^*^4^	*S. suis, S. agalactiae, S. pneumoniae, S. pyogenes*	*tet*(40), *tet*(O/W/32/O), *erm*(B), *ant*(6’)-Ia*, aph*(3’)	*rplL*	Tyrosine	[143, 150, 244]
ICE*Ssu*SC216^*2^	*S. suis*	*tet*(O), *erm*(B), *optrA, aadD*	*rplL*	Tyrosine	[97]
Tandem_ICE*Ssu*SC317^*2^	*S. suis*	*tet*(L), *tet*(O), *optrA*	*rumA*	SR	[97]
ICE*Ssu*BSB6^*2^					
	*S. suis*	*tet*(O/W/32/O), *erm*(B), *aph*(3’)-IIIa*, ant*(6’)-Ia*, sat4, vanG* operon	*rplL*	Tyrosine	[121]
ICE*Ssu*JH1308-2^*2^	*S. suis*	*tet*(M), *ant*(6’)-Ia	*rplL*	Tyrosine	[89]
ICE*Ssu*JH1301^*2^	*S. suis*	*tet*(O), *erm*(B), *aph*(3’)-III	*rplL*	Tyrosine	[89]
Tn*1549*					
dICE_Tn*1549_rplL*	*S. suis*	*tet*(O/W/32/O)	*rplL*	Tyrosine	[145]
ICE_Tn*1549_rbgA*	*S. suis*	*erm*(B)	*rbgA*	Tyrosine	[145]
Tn*1549*	*S. suis*	*vanB* operon	*hsdM*	Tyrosine	[89]
ICE*Sp*1108	*S. suis, S. agalactiae, S. pneumoniae,S. pyogenes*	*tet*(L), *tet*(W), *erm*(B), *lnu*(B), *erm*(TR), *ant*(6’)-Ia, *cat*	*rumA*	Tyrosine	[89]
*vanG*					
ICE_*vanG*_*lysS*	*S. suis*	*tet*(W)	*lysS*		[145]
Tn*GBS2*					
ICE_Tn*GBS2*	*S. suis*, *S. agalactiae*	*tet*(L), *aph*(3’)-IIIa, *ant*(6)	Diverse insertion sites, but not *rplL*	Tyrosine	[145, 245]
ICE*SsuTYPE3_rplL*	*S. suis, S. agalactiae, S. pneumoniae, S. pyogenes*	*tet*(L), *tet*(W), *erm*(B), *lnu*(B), *ant*(6’)-Ia*, cat*	*rplL*	Tyrosine	[89]
IMEs					
Integrated in MGEs					
IME_PPI^*3^	*S. suis, S. thermophilus*	*tet*(O), *tet*(40), *erm*(B)	*PPI*	SR	[145]
IME_SNF2^*3^	*S. suis*	*tet*(O), *erm*(B)	*SNF2*	SR	[145]
Integrated in chromosome					
IME_*Ssu*NSUI181_*tRNALeu*	*S. suis*	*aph*(3’)-IIIa, *ant*(6)-Ia, *sat4, vanZ*	*tRNA-leu*	Tyrosine	[145]
IME_*Ssu*NSUI231_*rpsI*	*S. suis*	*tet*(O)	*rpsI*	Tyrosine	[145]
IME_HTH-XRE-reg	*S. suis*	*ant*(6)-Ia	HTH-XRE regulator	SR	[145]
Prophages					
Prophage_*rumA*	*S. suis*	*tet*(O/W/32/O), *tet*(W), *erm*(B), *lnu*(B), *lnu*(C), *lsa*(E), *mef*(A), *ant*(6’)-Ib, *aph*(3’)-IIIa, *ant*(9’), *sat4*	*rumA*		[145]
ΦSC181	*S. suis*	*mef*(A), *aacA, aphD, cat, optrA*	*rumA*		[97]
Φm46.1^*4^	*S. suis*, *S. pyogenes*	*tet*(O), *mef*(A)	*rumA*	Site-specific recombinase	[89, 97]
ΦSsu1135/10	*S. suis*	*erm*(B), *ant*(6’)-Ia, *aph*(3)-IIIa	*rumA*		[170]
ΦSsUD.1	*S. suis*, *S. pyogene*s	*tet*(W), *erm*(B), *ant*(6’)-Ia, *aph*(3’)-III*, sat4*	*rumA*		[89, 97, 170]
ΦJH1301	*S. suis*	*mef*(A), *ant*(6’)-Ia*, cat, sat4*	*cysM / rumA /* SSU1958		[89]

ICEs, IMEs, and prophages are listed including the identified AMR genes, integration site, and integrase family (if known). ICEs are organized in families according to their conjugation module, and IMEs are grouped into two categories (indicated in bold). The presence of these elements in other bacterial species is notified. The table is not intended to be exhaustive but illustrative; for further information, see Bellanger et al. [144]

MGEs, mobile genetic elements; AMR, antimicrobial resistance; ICEs, integrative and conjugative elements; *llmO*, luciferase-like monooxygenase gene; SNF2, SNF2-family helicase; *rplL*, 50S ribosomal protein L7/L12; *rum*, RNA uracil methyltransferase; *mutT*, MutT/NUDIX hydrolase-family protein; *rbgA*, ribosome biogenesis GTPase A; dICE, derivative ICE; SR, serine recombinase; *hsdM*, type I restriction system adenine methylase; *rplU*, 50S ribosomal protein L21; IMEs, integrative and mobile elements; PPI, peptidyl-prolyl isomerase; *rpsI*, 30S ribosomal protein S9; HTH_XRE, helix-turn-helix XRE-family-like proteins; *cysM*, cysteine synthase.^*1^ICEs belonging to sub-family Tn*5252*. ^*2^ICEs belonging to sub-family ICE*Sa*2603. ^*3^MGEs studied for transfer between bacteria. *erm*(TR) genes include erm(B), erm(T) and an erm(A) subclass^*4^Prophage Φm46.1 is located in *S. suis* in tandem with several ICEs but can also behave as an independent element [89].

*S. suis* IMEs show more diversity than ICEs. IMEs are highly abundant in streptococcal species. Actually, IME-related elements (*n* = 457) were more prevalent than ICE-related elements (*n* = 377) in a large panel of *S. suis* genomes [145]. They can harbor canonical relaxases of the MOBC, MOBV, and MOBQ families or putative non-canonical relaxases with various domains [145]. On the *S. suis* chromosome, IMEs can be integrated in various genes (Table 2), including the tRNA-Leu- and tRNA-Asn-encoding genes, a putative peptidyl-prolyl isomerase-encoding gene (*PPI*), SNF2, and the house-keeping genes *rpsI*, *rpmG*, *guaA*, and *traG*, amongst others (Table 2). Some of these genes are located within ICEs of the Tn*5252* family (e.g. *PPI* and SNF2). As ICEs, IMEs can carry AMR genes. Notably, 80% of the AMR genes in 247 *S. suis* genomes were present within the IMEs, whilst 20% were within the ICEs, which indicates the importance of IMEs as antibiotic-resistance gene dispersers. Most of these IMEs are carried by ICEs, mainly of the Tn*5252* family (for further information [53]).

### Transformation

*Streptococcus* species can acquire genes by direct uptake of extracellular DNA (eDNA). This process requires transport of the DNA into the cell and its integration into the chromosome by homologous recombination. The genes involved are regulated by quorum sensing. *S. suis* employs the ComRS system. The *comS* gene encodes a precursor peptide that is proteolytically processed into the pheromone XIP, which is secreted into the extracellular environment. ComR is a cytoplasmic transcriptional activator. When XIP accumulates in the environment at high cell density, it is taken up into the cells by an oligopeptide-permease (Opp), where it binds, and thereby activates ComR. Activated ComR induces the expression of *sigX*, which encodes an alternative sigma factor, and its regulon [151]. SigX stimulates the transcription of the competence genes encoding the DNA-uptake machinery, known as transformasome, which is a T2SS-like machine and consists of a pilus, an endonuclease called EndA, and the DNA transport proteins (ComEA, EC, FA). Based on experimental evidence in the pneumococcus, *S. suis* pili probably function as DNA receptor on the cell surface [152]. After binding the pilus, the pilus retracts, and ssDNA crosses the membrane through the transformasome.

There are three types of ComRS systems in streptococci [151], referred to as type I to type III. The distinction is based on differences in the sequence of the XIP produced and in the C-terminal domain of ComR with which XIP interacts [153]. Each XIP is specific to a group of *S. suis* strains [154], as cognate XIP-ComR interaction is required to activate the system [153]. *S. suis* elements for transformation share high similarities with those of other streptococcal species [154]. Natural transformation is stimulated by environmental stresses, such as starvation or the presence of antibiotics, as well as the presence of active porcine or human sera [154].

Natural transformation requires chromosomal DNA release. It is assumed that eDNA is released by bacterial cell lysis facilitated by the expression of autolysins, such as LytA, LytB, and AtlI. These enzymes are located at the cell surface and cleave covalent bonds in the cell wall. The cell wall consists of a network composed of polymeric chains of *N*-acetylglucosamine-β-(1,4)-*N*-acetylmuramic acid interconnected via short peptides bound to the lactyl group of *N*-acetylmuramic acid. LytA is an *N*-acetylmuramoyl-L-alanine amidase that hydrolyzes the cell wall by cleaving the lactyl-amide bond. LytA does not cause cell lysis during normal growth [155], but it induces cell lysis during stationary phase or when cell-wall synthesis is disrupted by antibiotic treatment or nutrient depletion. LytB is an endo-β-*N*-acetylglucosaminidase that cleaves the β(1,4) glycosidic bond between *N*-acetylglucosamine and *N*-acetylmuramic acid. LytB has a role during bacterial cell division, acting as a chain-dispersing enzyme during the separation of daughter cells [156]. AtlI contains a catalytic *N*-acetylmuramoyl-L-alanine amidase domain and induces bacterial autolysis. SigX controls a competence-induced cell-lysis mechanism called fratricide. Competent cells stimulate lysis of non-competent cells in the same ecological niche, which leads to the release of chromosomal DNA into the milieu. In this process, the murein hydrolase CrfP is an important player. CrfP is exported to the extracellular milieu and lyses *S. suis* [157]. An immunity protein encoded by the *comM* gene is produced by competent cells and protects them from their own lysins [158]. Apart from PG hydrolases, small peptide bacteriocins, called suisins if produced by *S. suis*, mediate antagonistic activities to different bacteria, ultimately leading to lysis and DNA release. The two-peptide suisin CibAB participates in this process. Because the DNA released from lysed cells can be taken up by competent attacker cells, the rate of gene transfer is greatly increased. Three suicin synthesis clusters have been described, i.e., those for suicins 65, 90–1330, and 3908 [159–161]. Interestingly, they can be located on ICEs. Thus, suicins can provide an advantage to ICE-carrying strains by inactivating competitors, but they can also enhance the uptake of novel AMR genes by stimulating the presence of eDNA.

The exchange of AMR genes in *S. suis* by natural transformation has experimentally been demonstrated. Recently, Yu et al. [162] identified the genomic island *SsuSC128* in *S. suis* strain SC128. This non-mobilizable island carries *tet*(L), *tet*(M), and *catA8* conferring a tigecycline- and chloramphenicol-resistance phenotype [162]. *SsuSC128* was introduced into the genome of *S. suis* strain P1/7 by natural transformation, and the resulting transformants showed an increased MIC to tigecycline [162]. Thus, natural competence could mediate the transfer of non-mobilizable elements. This mechanism could be relevant, for example, for the acquisition of *pbp* gene variants that confer resistance to β-lactams. It is assumed that gene exchange by transformation has contributed significantly to the increasing incidence of penicillin-resistant *S. pneumoniae* [163, 164], and this is probably also the case in *S. suis*. In this context, the acquisition of a *pbp2x* gene of *S. pneumoniae* by a *Streptococcus mitis* strain has been reported [165]. Thus, exchange of *pbp* sequences could quickly generate β-lactam-resistant strains by introducing multiple simultaneously occurring substitutions in a single gene. This could explain the increasing β-lactam-resistance rates in *S. suis* reported in various countries over time (Figure 1). However, the rates of β-lactam resistance increase only slightly as compared to the rates of resistance to tetracyclines or lincosamides (Figure 1), the genetic determinants for which are located on conjugative elements (Table 2). This supports the notion that AMR gene transfer via transformation occurs less frequently than via conjugation. This difference in frequency could explain the large differences in resistance rates for different antibiotic families (Figure 1). Also, *S. suis* genomes contain AMR genes located on defective ICEs and IMEs whose genes involved in excision and/or conjugation are truncated. Up to 40 ICEs and 45 IME derivatives that lack a relaxase gene cluster were found in 215 *S. suis* genomes [145]. Therefore, these elements are not mobilizable by conjugation. The abundance and spread of these elements in *S. suis* genomes of different lineages could be explained by alternative transfer systems, such as natural transformation.

### Transduction

Transduction is the mechanism by which DNA is transferred between bacteria via bacteriophages (a.k.a. phages). Transduction can take place by three mechanisms, called generalized, specialized, and lateral transduction. In generalized transduction, bacterial DNA can be randomly packed into new phage particles during the lytic cycle and then be transferred into a new host. Some MGEs hijack the phage DNA-packing machinery for their own transfer [166]. In specialized transduction, only bacterial DNA directly adjacent to the prophage is packed into new virus particles as result of anomalous prophage excision. In lateral transduction, phage DNA replication is initiated in the integrated prophage and results in the amplification also of flanking host DNA. The amplified DNA is packed while still integrated in the chromosome until the virus capsule is full. In this way, long fragments of the host genome, including MGEs or independent AMR genes, are packed into phage particles and transferred to a new host. The exchange of AMR genes occurs at higher frequencies by lateral transduction than by generalized and specialized transduction [167].

The ubiquity and high abundance of phages in nature suggests that transduction occurs at high frequency. Table 2 lists prophages described in *S. suis* carrying AMR genes. Phages are often specific for a certain species or even for strains of a species, as the phage receptor and bacterial defense mechanisms can vary. Nevertheless, the exchange of AMR genes between different streptococcal species by transduction has been reported. There is in vitro evidence of transfer of bacteriophage Φm46.1 between *S. suis* and *S. pyogenes.* Interestingly, the transfer occurs in both directions, but the transfer rate is higher from *S. suis* to *S. pyogenes* than vice versa (8.5 × 10^–4^ versus 2.3 × 10^–9^) [168], again supporting the notion that *S. suis* is a reservoir of AMR genes. This phage is one of the most frequently mobile elements found in *S. suis* isolates and carries the resistance genes *mef*(A) and *tet*(O) [169], conferring resistance to erythromycin and tetracycline, respectively. It also improves *S. suis* fitness and leads to overexpression of *atlI*, although the latter effect was not observed in other *Streptococcus* species [168]. *S. suis* strain SC181 contains a Φm46.1-like prophage (Table 2), named ΦSC181, which carries many AMR genes including, for example, an *optrA* gene [97] (Table 2). ΦSsu1135/10 was found in *S. suis* isolates from Brazil [170] (Table 2). This phage carries *aph*(3’)-IIIa, *ant*(6’)-Ia, and *erm*(B) resistance genes [170], indicating that it can spread several AMR genes turning infected bacteria into multidrug resistant strains. Another phage, named ΦSsUD.1, contains *tet*(W), *aph*(3’)-III, *ant*(6’)-Ia, *aphA3*, *aadE*, *sat4*, and *erm*(B) [170], conferring a macrolide–aminoglycoside–streptothricin-resistance phenotype [171], as well as a *cadC/cadA* cadmium-efflux cassette [142]. All these bacteriophages are like each other and are found in different species of *Streptococcus*. Together, these data support the notion that AMR genes can also be spread in *S. suis* by transduction and that this mechanism can contribute to a rapid acquisition of a multidrug-resistant phenotype.

### Alternative HGT mechanisms

Extracellular vesicles are membrane-derived lipid bilayers released into the milieu by Gram-negative and Gram-positive bacteria. They often show a typical spherical morphology of 20 to 300 nm in diameter. The composition of extracellular vesicles of Gram-positive bacteria resembles essentially that of the cytoplasmic membrane; however, they are enriched in anionic phospholipids and may contain a variable composition of lipoproteins [172]. Extracellular vesicles can encapsulate different molecules, including cytosolic proteins, secreted proteins, and nucleic acids. Proteins can be localized in the vesicle lumen, integrated into the vesicle membrane, or membrane anchored and exposed at the vesicle surface. Genetic material can be present in the vesicle lumen or externally associated with the membrane. Extracellular vesicles are used to interact with eukaryotic and prokaryotic cells in the environment without establishing direct cell-to-cell contact. They can deliver their cargo to eukaryotic cells through three pathways, (i) endocytosis, (ii) membrane fusion, and (iii) clathrin-dependent endocytosis. In many pathogenic bacteria, extracellular vesicles can act as carriers of virulence factors, such as toxins [173], or of factors mediating evasion of the host immune system [174]. They can also participate in interbacterial interactions [175] by carrying quorum-sensing molecules [176] or toxins directed to other bacteria [177], and by stimulating the formation of bacterial associations, such as biofilms [178]. Extracellular vesicles can also deliver chromosomal DNA or plasmids, and, indeed, they have been associated with HGT in Gram-negative bacteria, including in AMR gene transfer. For example, *Acinetobacter baumannii* can acquire resistance to carbapenem antibiotics via extracellular vesicles harboring the *bla*_*OXA-24*_ gene [179]. In contrast to transformation, where bacterial lysis is required, vesicles are released from live bacteria, which can modulate vesicle production according to environmental conditions [180]. Furthermore, in contrast to free DNA involved in transformation, vesicle-encapsulated DNA is protected against extracellular nucleases, which can be present in host tissues. Thus, this process of HGT confers a particular advantage for the dissemination of genes and constitutes an alternative and more secure way of dispersing AMR genes in vivo. Although this mechanism has yet to be proven in Gram-positive bacteria, DNA is an abundant constituent of extracellular vesicles of these bacteria [181]. Thus, AMR-gene transfer via extracellular vesicles could occur in Gram-positive bacteria, a hypothesis that requires experimental confirmation.

Many streptococcal species, including *S. suis*, were reported to produce extracellular vesicles. As compared with the cytoplasmic membrane, vesicles produced by *S. pneumoniae* are enriched in lipoproteins and short-chain fatty acids [182]. In some streptococcal species, the protein composition of the vesicles varies and also deviates from that of the cytoplasmic membrane as it contains only few membrane proteins [183]. The production and size of extracellular vesicles vary between strains and also fluctuate depending on the growth conditions [184]. For example, the extracellular vesicles of *S. pneumoniae* strain R6 are 130–160 nm in diameter [185], while those of strain TIGR44 are between 25 and 250 nm [183]. The extracellular vesicles of *S. suis* strain P1/7 range in diameter up to 130 nm [186]. Proteomic analysis of these vesicles identified up to 46 different proteins, including cytoplasmic, membrane/cell wall-associated, and secreted proteins; nine of them were virulence factors. The sorting mechanisms used to load the protein cargo of these vesicles have not been elucidated. Also in pathogenic streptococci, extracellular vesicles have an important role during bacterial infection. In pneumococci, they stimulate proinflammatory immune responses and antigen presentation by eukaryotic cells [185], and they activate C3b deposition and the formation of the membrane attack complex. Furthermore, they bind factor H and decrease bacterial opsonophagocytosis [183]. Extracellular vesicles of *S. suis* degrade neutrophil extracellular traps (NETs), which contributes to the evasion of the immune response, and they activate the nuclear factor-kappa B signaling pathway in monocytes and macrophages, which increases the permeability of the blood brain barrier [186]. So far, there are no reports of gene transfer mediated by extracellular vesicles in *S. suis* or any other Gram-positive bacteria. However, genetic material has been reported to be associated with streptococcal vesicles. Actually, in *S. mutants,* a major contributor to human dental caries, extracellular vesicles form an active route for the delivery of eDNA to the extracellular matrix (ECM) of the biofilm [181]. Interestingly, β-lactamase-positive *Moraxella catarrhalis* produce extracellular vesicles that carry β-lactamase, which protects *S. pneumoniae* against β-lactams [187]. Very likely, *S. suis* may also benefit of antibiotic-degrading enzymes present in extracellular vesicles produced by other AMR bacteria. Yet, this mechanism must be demonstrated.

## Role of *S. suis* biofilms in antimicrobial resistance and microbial persistence

The formation of biofilms increases tolerance and resistance of bacteria to antimicrobials. This feature of biofilms is caused by several mechanisms [188] that vary between bacterial species and even between strains and ultimately depends on (i) the dynamics of biofilm formation, (ii) biofilm architecture, and (iii) ECM composition.

### Biofilm-mediated antibiotic tolerance

Biofilm formation is initiated by the binding of planktonic bacteria to a substratum, an activity mediated by long appendices that protrude from the cell surface, including flagella and fimbriae or pili. *S. suis* can produce peritrichous and flexible fimbriae [189, 190], but they do not produce flagella. In the three major human streptococcal pathogens, i.e., *S. agalactiae*, *S. pyogenes*, and *S. pneumoniae,* a role for pili in biofilm formation has been reported, but their function has not been examined yet in *S. suis*. After binding to the substratum is established, shorter surface-exposed structures, such as fibrinogen-binding proteins, establish additional contacts and stabilize the binding. The proteins contain particular motifs that mediate adherence to different host structures or promote interbacterial interactions [191]. However, the binding of cell-surface-exposed proteins to the substratum or to the ECM can be occluded in many bacteria by capsule. This was also demonstrated for *S. suis* [192]. Thus, the loss of capsule enhances biofilm formation and, consequently, antibiotic tolerance. After establishing intimate contact with the substratum, bacteria proliferate to form microcolonies, i.e., small aggregates of bacteria originating from the same parental cell, and secrete ECM that stabilizes cell-to-cell and cell-substratum interactions. Microcolonies can interact with each other to form macrocolonies. At the end, the spatial organization of the biomass on the substratum defines the biofilm architecture, which varies during biofilm development. In general, mature biofilms are more resistant to antibiotics than younger biofilms [193] and, thus, the level of tolerance to antibiotics varies according to biofilm dynamics.

Electron microscopic examination of *S. suis* biofilms formed on abiotic surfaces revealed interconnected aggregates of different sizes with well-defined intervenient spaces in between [192, 194]. These spaces could constitute channels for the exchange of molecules and resources within the biofilm community. This organization of the biomass generates gradients of dispersion that affect the distribution of antibiotics within the biofilms. Cells within clusters are exposed to lower antibiotic concentrations than those located in the upper layers of the biofilm. The dispersion gradients also limit the availability of nutrients and oxygen for bacteria and generate excess of waste products in the deeper layers. This causes starvation and hypoxia, which force bacteria to slow down their metabolism and growth, thereby entering into a quiescent state [195]. As a result, bacteria within a biofilm are metabolically heterogeneous. Quiescent-state bacteria are less susceptible to antibiotics because crucial processes targeted by antibiotics are stopped. Hence, binding of antibiotics to their targets does not exert bactericidal activity [196]. Nutrient starvation also activates the stringent response, a mechanism that reprograms bacterial metabolism. To save energy and nutrients, the stringent response decreases RNA synthesis [197]. It is signalled by the alarmone (p)ppGpp [197, 198]. In *S. suis*, (p)ppGpp is synthesized by two different proteins, RelA and RelQ [197, 199]. This system seems to play a key role in *S. suis* pathogenesis, since it regulates the expression of genes presumably coding for virulence factors, such as *cps2,* a member of the capsule operon, and *eno*, which encodes the adhesin enolase, amongst others. Together, down regulation of capsule synthesis and over expression of adhesins, improve the adhesion to and invasion of host epithelial cells [200]. The response induced by (p)ppGpp helps *S. suis* to evade phagocytosis by host macrophages [199], presumably by promoting biofilm formation [198]. The involvement of the stringent response in antibiotic tolerance and resistance has been shown in many bacteria (reviewed in Hobbs and Boraston [201]). The mechanisms behind seems to be related to the upregulation, downregulation, or mutation of stress-responsive genes [201]. In *S. suis*, the activity of pyruvate dehydrogenase, which is encoded by the *pdh* gene and whose expression is upregulated by (p)ppGpp, affects adhesion to and invasion of host epithelial tissues as well as biofilm formation and stress response [202, 203]. Pyruvate dehydrogenase activity is suspected to increase antibiotic tolerance by increasing biofilm production [202].

Variations in the biofilm structure influence antibiotic tolerance and resistance. In many bacteria, large inter-strain variations in biofilm structure have been observed, and they appear to be a consequence of variation in the expression of surface-exposed proteins or ECM components. Presumably, this occurs also in *S. suis*. Metagenomic analysis of 375 *S. suis* genomes revealed that the species is genetically and phenotypically highly heterogeneous [204], and this could be translated in variation in biofilm-forming capacities. In fact, the biofilm-forming capacity of 46 clinical *S. suis* isolates showed high variability even between strains of the same serotype, while comparison of biofilm architecture also revealed large differences [194]. Thus, it is probable that the antibiotic-tolerance capacity in *S. suis* is strain dependent.

The ECM composition impacts antibiotic efficacy. ECM may contain extracellular polysaccharides, DNA and RNA, proteins, membrane vesicles, and cell debris. ECM components can interact with certain antibiotics, thereby affecting antibiotic effectivity. Extracellular polysaccharides are relevant constituents of the ECM from many bacterial species. Their nature is diverse, including linear or branched polysaccharides, and varies between bacterial species. Notably, the presence of unstructured polysaccharides, a.k.a. slime, in the ECM can strongly affect biofilm tolerance to antibiotics. An example is alginate produced by *P. aeruginosa*, which protects biofilms from aminoglycosides [205]. Alginate is negatively charged and, thus, it may interact with positively charged antibiotics. The production of extracellular polysaccharides was independently demonstrated in biofilms of *S. suis* by two research groups using microscopy. The presence of extracellular polysaccharides was visualized in biofilm cells, but not in planktonic cells of *S. suis* strain NJ-3 by FITC-ConA staining [194]. Slime was also visualized in biofilms of *S. suis* strain 95–8242 using Congo red staining and scanning electron microscopy [205]. Remarkably, biofilms of *S. suis* strain 95–8242 were 1000 times more tolerant to penicillin G and ampicillin than planktonic cells of this strain, but such differences were much smaller in the case of *S. suis* strain AAH4, which does not produce extracellular polysaccharides [205]. *S. suis* exopolysaccharides could interact with penicillin G and ampicillin and, thus, deplete antibiotic activity, a hypothesis that has not been explored. However, the production and the structure of these polysaccharides seem to vary between strains, consistent with the large variability in the genes coding for polysaccharide synthesis. In addition to exopolysacharides, eDNA is a relevant component of the ECM in many bacteria, and it is definitively more conserved than the exopolysaccharides. eDNA plays an important role in promoting and modulating biofilm development by participating in the adhesion to the substratum and in the structural integrity of biofilms. Also, eDNA can enhance resistance to antibiotics. In *Staphylococcus epidermidis*, eDNA enhances tolerance to vancomycin about 100-fold [206]. The binding constant of vancomycin to DNA is 100-fold higher than that to its target [206] and, thus, eDNA may trap this antibiotic in the ECM. The presence of eDNA in the ECM of biofilms was reported for several pathogenic streptococci, including *S. pneumoniae* [207], *S. mutants* [208], and *S. intermedius* [209]. For *S. suis*, only biofilms formed in the presence of neutrophils [210] have been reported to be sensitive to DNase. In this special case, eDNA is released from neutrophils to entrap bacteria in NETs, an immune defence mechanism. eDNA can also be released from bacteria by a specific DNA secretion system, but such system is not present in *S. suis*. So far, there is no direct evidence that *S. suis* actively secretes DNA. Alternatively, eDNA can be released from extracellular vesicles or by bacterial autolysis, which is enhanced by overproduction of autolysins that form part of the cell wall synthesis machinery. Two *S. suis* autolysins (AtlAss and AtlI) have been reported to contribute to biofilm formation [211, 212]. Also, antibiotics can promote the release of eDNA and increase biofilm production and antibiotic resistance. Indeed, subinhibitory concentrations of amoxicillin, lincomycin, and oxytetracycline were reported to enhance biofilm formation in *S. suis* [213]. Yet, experimental evidence is required to demonstrate that eDNA contributes to the ECM of *S. suis* as has been described for other streptococci. Yet, another component of the ECM of *S. suis* biofilms is fibrinogen. Fibrinogen is a mammalian protein present in blood and host tissues. Fibrinogen binds to fibrinogen-binding proteins exposed at the bacterial cell surface, resulting in cell aggregation [214], which ultimately stimulates biofilm formation. This can be relevant during bacteraemia, in which fibrinogen can generate bacterial aggregates that are more resistant to phagocytosis and allow bacteria to adhere to organs and tissues, causing endocarditis or arthritis. Interestingly, fibrinogen-stimulated *S. suis* biofilms showed higher minimal bactericidal concentrations to penicillin G than planktonic cells [214].

Intercellular communication contributes to antibiotic tolerance and resistance of biofilms through multiple mechanisms. In general, biofilms formed by mutants in quorum-sensing systems are less tolerant to antibiotics as has been reported for *P. aeruginosa* [215] and *S. aureus* [216], amongst others. In *S. suis*, quorum sensing also regulates biofilm formation and influences thereby antibiotic tolerance. Addition of autoinducer-2 (AI-2) (2 µM) to *S. suis* cultures increased biofilm formation [217], while deletion *luxS*, involved in the synthesis of AI-2, reduced biofilm formation in the presence of fibrinogen [218]. This is in agreement with a reduced expression of fibrinogen-binding proteins in the *luxS* mutant [218]. Thus, considering that the biofilm biomass and architecture are important factors for antibiotic tolerance, AI-2 considerably contributes to the development of tolerance. In contrast, high concentrations of AI-2 (> 5 µM) reduced biofilm formation and growth in *S. suis* [217]. High concentrations of AI-2 trigger a signalling cascade that leads to the expression of the *tet*(M) gene on Tn*916*, resulting in resistance to tetracycline [105], which is an antibiotic-resistance mechanism independent of the tolerance generated by the biofilm. Expression of *tet*(M) is also activated by tetracycline at concentrations below the minimal bactericidal concentration [105], conditions that can also be met inside biofilms. In addition, LuxS/AI-2 is involved in fluoroquinolone resistance by regulating the synthesis of efflux pump SatAB [135, 137]. Together, these data show that quorum sensing directly contributes to antibiotic resistance by inducing expression of AMR genes and indirectly to antibiotic tolerance by regulating biofilm formation and biofilm biomass. Apart from quorum sensing, other intercellular communication systems have been described in *S. suis*. As described above, some *S. suis* strains produce suicins that target competing bacteria [161, 219]. Killed target bacteria release intracellular components, including DNA, which can be taken up by transformation but also contribute to biofilm formation and antibiotic tolerance. To summarize, biofilm formation in *S. suis* causes tolerance to antibiotics through biofilm architecture, ECM composition, and intercellular communication.

### Biofilm-mediated antibiotic resistance

Biofilm formation drastically enhances HGT and, consequently, the transfer of resistance genes. This is caused by several mechanisms. Firstly, biofilms can be formed by a diversity of bacterial species that establish close proximity. *S. suis* can form mixed biofilms with other species, as was shown, for example, in vitro with *Actinobacillus pleuropneumoniae* [220], another pathogen of the upper and lower respiratory tract of pigs. Such mixed biofilms increased the expression of virulence factors in both species and enhanced antibiotic resistance. Secondly, the reduced mobility of cells in biofilms and the high bacterial density favour interbacterial interactions. This facilitates the conjugation process, which is extensively used by *S. suis* for gene transfer. Conjugative transposons of the Tn*916* family carrying genes coding for tetracycline resistance were shown to be transferred within multispecies biofilms of oral bacteria [221]. Thirdly, the presence of eDNA and extracellular vesicles within the ECM facilitates the transformation process. Transformation and the presence of extracellular vesicles have been demonstrated in *S. suis*. This could facilitate the dispersion of mutant alleles, such as those for DNA gyrase and PBPs, or of genes coding for antibiotic-degrading enzymes.

Biofilm formation enhances the mutation rate through multiple mechanisms. Exposure to agents that elicit oxidative stress increases the mutation rate. Oxidative stress is related to the generation of reactive oxygen species, which cause direct DNA damage and mutations. At sublethal doses of antibiotics, as can be present within the biofilm mass, the production of reactive oxygen species promotes antibiotic resistance by triggering the production of efflux pumps. Increased mutation rate can be a consequence of the activation of the SOS response. The SOS response is activated when bacterial DNA is damaged, a condition that occurs at high frequency within certain parts of the biofilm. RecA binds to damaged DNA and stimulates self-cleavage and, thereby, inactivation of the repressor LexA. This promotes the expression of SOS genes, the products of which repair the DNA, enhance mutagenesis, and slow down bacterial growth until the DNA is fixed.

The SOS response, especially in combination with a lack of amino acids such as lysine, leucine, and cysteine, induces a high tolerance to ofloxacin [222]. Interestingly, the SOS response enhances the transfer of ICEs [223], which, as described above, is a prime mechanism in *S. suis* for the exchange of resistance genes. Mutations can occur in genes coding for the mismatch repair system, which increases the mutation rate then by about 100- to 1000-fold. This hypermutator phenotype has been reported in several pathogenic bacteria, including *P. aeruginosa, Haemophilus influenzae*, and *S. aureus* [224], but not yet in *S. suis*. If the mutation rate is indeed increased in *S. suis* biofilms, resistance to β-lactams, fluoroquinolones, and aminoglycosides could be enhanced by favouring mutations in PBP-encoding genes, the *parC* and *gyrA* genes, and the *rpsL* gene, which codes for 16S rRNA. Also, the enhanced mutation rate may increase the production of efflux pumps or alter the specificity of enzymes of the folate-synthesis pathway. However, an early study in *S. mutans* proved that the accumulation of mutations in biofilms was associated with natural selection rather than with a high mutation rate [225]. This raises the question if hypermutability is favoured within *S. suis* biofilms. Overall, mechanisms of biofilm-mediated antibiotic resistance in *S. suis* involve increased HGT and intercellular communication.

## Concluding remarks

For more than three decades, antibiotics have been used to control *S. suis* infections. Undoubtably, they have saved thousands of pigs and even human lives, particularly in view of the expansion of the intensive pig production industry. Their efficacy has, however, declined by the successive acquisition of resistances. High AMR rates (> 80%) to lincosamides, macrolides, and tetracyclines were reported over the world (Figure 1, panels A-C). Even further, this has been accompanied by a rapid development of multidrug resistance over time (Figure 1D). The genetic origin of AMR has been related to mutations in the target site, the production of target-protective and antibiotic-degrading or -modifying enzymes, and of antibiotic exporters (Table 1), whose genes are spread by HGT. Massive metagenomic analysis of resistant isolates showed the location of most of the corresponding AMR genes in MGEs, such as ICEs, IMEs, and derivatives (Table 2). These elements can carry genes for autonomous conjugation and integration of the MGE into the bacterial chromosome. Conjugation seems a very efficient way for gene dispersion in *S. suis*, as judged by the massive presence of MGEs in *S. suis* genomes of different genetic lineages. Examination of streptococcal genomes showed a higher accumulation of these elements in *S. suis* than in other pathogenic *Streptococcus* species. Therefore, *S. suis* is considered as an AMR gene reservoir capable of accumulating and transferring MGEs at high frequency. Remarkably, some MGEs carry AMR genes for several different antibiotics leading to a rapid increase of multidrug-resistant strains. Yet, resistance to β-lactams, quinolones, and, in some regions, amphenicols remains low (Figure 1, panels A–C). Therefore, they are the first choice of drug to treat streptococcal swine disease in many countries nowadays. The slow development of resistance to these antibiotics contrasts to that reported for lincosamides, macrolides, and tetracyclines (Figure 1, panels A–C). This is in part due to a lower consumption of these antibiotics and/or their mechanism of resistance. For example, the main mechanism of β-lactam resistance is polymutation in *pbp* genes [62, 65]. *S. suis* could also transfer *pbp* resistance alleles by HGT, but, as they are not located in MGEs, gene transfer must be mediated by natural transformation (or other methods alternative to conjugation and transduction). Natural transformation is an inducible mechanism triggered by the quorum-sensing system, diverse environmental factors, and, possibly, unknown host factors [154, 226]. Ultimately, it depends on ComR-XIP interaction [153], which is strain specific. Thus, these possible limitations could explain in part the low rates of resistance to certain antibiotics.

Clearly, *S. suis* infection requires alternatives to conventional antibiotics. The understanding of the mode of action of antibiotics, as well as of the mechanisms of resistance, can help in designing novel molecules or combinations of molecules to counteract the bacterial resistance mechanisms. Different platforms for the discovery of novel molecules or the improvement of the current ones have been proposed [227], and they should be urgently exploited. However, this report illustrates that *S. suis* can rapidly acquire resistance to antimicrobials, which results in a lack of economic appeal for pharmaceutical companies to invest in the development of new molecules, which could be out of the market within a few years. An alternative approach is the exploitation of plant extracts that contain bactericidal components as part of the native immune system of plants. Some examples are extracts obtained from red thyme, common thyme, oregano, and cinnamon, which showed bactericidal activity in vitro against virulent strains of *S. suis* [228]. Particularly, these extracts could be presented to piglets as feed additives, thereby preventing *S. suis* colonization, a crucial step in the infection process. However, natural extracts exhibit drawbacks for their application in vivo, including toxicity at the required concentrations or activity against beneficial commensal flora. Their application is poorly regulated by law, and their presence in the final meat product is unknown. Phage therapy, i.e., the use of phages that kill bacteria, is becoming again popular these years. Phages are easy to produce, specific, self-controlled, and they do not generate side effects in the host. Several phages that kill *S. suis* have been isolated [229]. Yet, phages have several disadvantages, i.e., they are strain specific, and bacteria can easily develop resistance, for instance by altering the phage receptors by mutation. Moreover, while phage receptors are fully available in planktonic cells, their accessibility in biofilms is compromised by the ECM, preventing the phages of reaching their targets. Also, the ECM of biofilms contains phage receptors that compete with those located at the bacterial cell surface. To overcome these limitations of phage therapy, phage-produced enzymes, required for membrane destabilization and bacterial lysis, have been proposed to treat *S. suis* infections [230, 231]. Positive results of this approach have been shown in experimental infections of laboratory animals. Yet, their high production costs remain a major concern.

Vaccination could also be a solution. Alternatives to bacterins have been proposed, for example live-attenuated or subunit-based vaccines. Several live-attenuated vaccines have been studied, including the use of mutants auxotrophic for aromatic amino acids [232], or mutants lacking virulence factors [233, 234]. In general, live-attenuated vaccines show better efficacy than bacterins because the native structure of antigens is retained, and the immune system is better stimulated. However, toxicity of certain components is a drawback. Also, considering the variety of HGT mechanisms in *S. suis* discussed here, there is a risk that the attenuated strain turns virulent by acquiring genes from other circulating strain in the pigs. In contrast to live attenuated vaccines, subunit-based vaccines are safe because of the purity of the antigens. The capsule has been studied as a vaccine candidate for specific serotypes, but it elicits a T-cell-independent response that is very limited in piglets. In human vaccines against *S. pneumoniae,* conjugation of the capsule to protein antigens carrying T-cell epitopes makes them more immunogenic and capable of eliciting T- and B-cell responses. Yet, this strategy greatly increases the cost of a porcine vaccine, particularly when multiple capsules must be included to elicit cross-protection against different serotypes, at least against the most prevalent ones (*n* = 9) [235]. Subunit vaccines based on proteins constitute a more promising approach as they can elicit T-cell-dependent responses. Many antigens were proposed as *putative vaccine candidates* and, indeed, some elicited protective immune responses against the homologous strain in experimental animals. Some examples include Antigen One (Sao), enolase (Eno), peptidase SsPepO, DNA nuclease SsnA, and IgA1 protease, as discussed in [3]. Results are promising but not enough to guarantee complete coverage of the circulating *S. suis* strains. Also, some antigens elicit a protective response in mice but not in pigs [3]. This suggests that better adjuvants could be very helpful to increase the efficacy of such vaccines. However, despite many trials and few patents, there are no commercial vaccine(s) on the market to effectively prevent the disease caused by the superbug *S. suis*.

## Supplementary Information

**Additional file 1.** **Measurement of antibiotic resistance and tolerance in *S. suis*.**

## Abbreviations

AACs: N-acetyltransferases
ABC: ATP-binding cassette
AI-2: autoinducer-2
AMR: antimicrobial resistance
ANTs: aminoglycoside O-nucleotidyltransferases
APHs: aminoglycoside O-phosphotransferases
Cats: chloramphenicol O-acetyltransferases
ECM: extracellular matrix
eDNA: extracellular DNA
EU: European Union
HGT: horizontal gene transfer
ICEs: integrative and conjugative elements
IMEs: integrative and mobilizable elements
MAMs: macrolide arrest motifs
MATE: multiple antibiotic and toxin extrusion
MFS: major facilitator superfamily
MGE: mobile genetic elements
MLS: macrolides, lincosamide, streptogramin
MLS_(B)_: macrolides, lincosamide, streptogramin B
Opp: oligopeptide permease
PBPs: penicillin-binding proteins
PG: peptidoglycan
RPP: ribosome-protection proteins
T4SS: type IV secretion system
