# Supplementary material for: How Streptococcus suis escapes antibiotic treatments
Source: Vet Res. 2022 Nov 12;53:91. doi: 10.1186/s13567-022-01111-3 (PMC9652813; doi:10.1186/s13567-022-01111-3)
Supplement: Supplementary file 1 — Additional file 1. Measurement of antibiotic resistance and tolerance in S. suis. [file 13567_2022_1111_MOESM1_ESM.docx]

**Supporting Information**

How *Streptococcus suis* escapes antibiotic treatments

Cristina Uruén, Carla García, Lorenzo Fraile, Jan Tommassen, and Jesús Arenas

**Measurement of antibiotic resistance and tolerance in *S. suis*.**

Antimicrobial susceptibility testing is an essential tool to select the most suitable antimicrobial for the treatment and to decrease the probability of selecting for antimicrobial resistance [1]. The most frequently used methods are the agar disc diffusion assay and the broth dilution assay, which are qualitative and quantitative techniques, respectively, and the E-test, which is a quantitative agar disc diffusion test. Nowadays, the most used quantitative technique is the broth microdilution test, which allows for determining the minimal inhibitory concentration (MIC) for each antimicrobial and each pathogen [2]. The MIC is defined as the lowest antimicrobial concentration that inhibits *in vitro* the growth of the target bacteria in specific conditions, usually 18 to 24 hours incubation in a culture medium at 37ºC starting from a standardized inoculum [3-5]. Obviously, these conditions are not similar to those in which bacteria grow *in vivo*, such as blood, extracellular fluid, or in the presence of pus or debris. Hence, the data obtained *in vitro* do not perfectly reflect what happens *in vivo*. In spite of these limitations, the MIC is the most frequently used pharmacodynamic parameter to determine the capacity of bacteria to resist antimicrobials [5-6]. One key point to obtain reliable data is that the method has to be performed by specialized laboratories using standardized methods, *i.e.* the methods from the Clinical and Laboratory Standards Institute (CLSI) [7]. Finally, the MIC obtained must be translated to antimicrobial resistance or susceptibility based on breakpoints that define from which concentration of an antibiotic a bacterial species is considered resistant or susceptible. If the MIC is less than or equal to the breakpoint, the bacteria are considered susceptible.

Several breakpoints can be used. Clinical breakpoints (CBPs) are used to categorize any bacterial strain as susceptible or resistant, *i.e.,* considering that the pathogen will most likely respond or not respond to therapy with the standard posology regimen, respectively. It is important to understand that CBPs for antimicrobials have been developed on the basis of an approved dosage regimen, which defines dose, frequency of administration, administration route, and duration of therapy [8]. Setting up CBP is complex because it is necessary to have MIC data distribution of the target bacterial species, pharmacokinetic data of the antimicrobial, data from clinical efficacy studies, and pharmacokinetic-pharmacodynamic analysis using Monte Carlo simulations as recently proposed Toutain, et al. [9]. Unfortunately, there is a scarcity of CBPs for *S. suis*. Only CBPs are available right now for ampicillin, penicillin, ceftiofur, enrofloxacin, florfenicol, and tetracycline, according to the latest CLSI recommendations [10, 11]. For antimicrobials for which CBPs are not available, diagnosticians may extrapolate data from one animal species to another or even from human data [12, 13]. Additionally, there is a surveillance system in many countries to monitor AMR, integrating data from humans, animals, food, and the environment. In food-production animals, isolates from clinical cases and commensals from healthy animals (available at slaughter) are assessed [14]. In this situation, epidemiological cut-off rather than CBPs is considered. Epidemiological cut-off is the MIC value that separates bacterial populations into those representatives of a wild-type population and those with acquired or mutational resistance to the drug. This is based on microbiological studies and does not necessarily indicate whether a drug will be clinically active, since it does not take into account what happens to an antibiotic within the body. Unfortunately, monitoring AMR of *S. suis* is not compulsory in many countries, but some surveillance systems gather information about AMR of this bacterium. However, not all surveillance systems are using the same breakpoint. For example, two of the best known surveillance systems in Europe (Resapath and Danmap) are using the qualitative agar disc diffusion method [15] and the quantitative broth dilution method [16], respectively, to determine AMR for *S. suis*.

It is important to note that genetically susceptible bacteria can survive transient exposure to bactericidal antibiotic concentrations through a phenomenon known as tolerance. Tolerance is defined as the ability, whether inherited or not, of microorganisms to survive transient exposure to high concentrations of an antibiotic without presenting a change in the MIC. Tolerance is assessed by the minimum bactericidal concentration (MBC), that is, the minimum concentration of antibiotic required to kill 99.9% of the cells [17]. This ability is usually acquired by a variety of mechanisms that are enhanced when the bacteria form biofilms. Biofilms are highly organized associations of bacteria, encased in an extracellular matrix (ECM) produced by themselves, that may be adhered to abiotic or biotic surfaces [18]. Biofilms contribute largely to bacterial tolerance and, additionally, they enhance resistance to antibiotics [19]. Indeed, *S. suis* forms biofilms with demonstrated tolerance to antibiotics [20, 21] (expanded in the section 5). In spite of a large amount of literature reporting the implication of biofilms on AMR and tolerance in many pathogens, there is not a single assay universally used to assess antimicrobial tolerance in *S. suis* biofilms. To address routine biofilm growth and susceptibility testing methods within laboratories, efforts were made to establish approved standard biofilm protocols ([www.astm.org](http://www.astm.org)). However, up to now, such methods are limited to those for the human pathogen *Pseudomonas aeruginosa*.

**References**

1. Fraile L. Antimicrobial therapy in swine: a practical approach. Zaragoza, Spain: Servet, 2013.

2. Richter A, Feßler AT, Böttner A, et al. Reasons for antimicrobial treatment failures and predictive value of in-vitro susceptibility testing in veterinary practice: An overview. *Vet Microbiol.*  2020;245:108694. doi:10.1016/j.vetmic.2020.108694.

3. McKellar QA, Sanchez Bruni SF, Jones DG. Pharmacokinetic/pharmacodynamic relationships of antimicrobial drugs used in veterinary medicine. *J Vet Pharmacol Ther.*  2004;27:503-14. doi:10.1111/j.1365-2885.2004.00603.x

4. Wayne PA. Performance standards for antimicrobial disk and dilution susceptibility tests for bacteria isolated from animals. 4th ed. *CLSI.*  2018.

5. Wayne PA. Performance standards for antimicrobial susceptibility testing. 28th ed. . *CLSI.*  2018.

6. Mouton JW, Vinks AA. Pharmacokinetic/pharmacodynamic modelling of antibacterials in vitro and in vivo using bacterial growth and kill kinetics. *Clin Pharmacokinet.*  2005;44:201-10. doi:10.2165/00003088-200544020-00005.

7. Wallmann J, Böttner A, Goossens L, et al. Results of an interlaboratory test on antimicrobial susceptibility testing of bacteria from animals by broth microdilution. *Int J Antimicrob Agents.*  2006;27:482-90. doi:10.1016/j.ijantimicag.2005.12.011.

8. Papich MG. Antimicrobials, susceptibility testing, and minimum inhibitory concentrations (MIC) in veterinary infection treatment. *Vet Clin North Am Small Anim Pract.*  2013;43:1079-89. doi:10.1016/j.cvsm.2013.04.005

9. Toutain PL, Bousquet-Mélou A, Damborg P, et al. En route towards European clinical breakpoints for veterinary antimicrobial susceptibility testing: a position paper explaining the VetCAST approach. *Front Microbiol.*  2017;8:2344. doi:10.3389/fmicb.2017.02344.

10. Wayne PA. Performance standards for antimicrobial susceptibility testing. 29th ed. *CLSI.*  2019.

11.Wayne PA. Understanding susceptibility test data as a component of antimicrobial stewardship in veterinary settings. 1st ed. *CLSI.*  2019.

12. Holmer I, Salomonsen CM, Jorsal SE, et al. Antibiotic resistance in porcine pathogenic bacteria and relation to antibiotic usage. *BMC Vet Res.*  2019;15:1-13. doi:10.1186/s12917-019-2162-8

13. Petrocchi-Rilo M, Martínez-Martínez S, Aguarón-Turrientes Á, et al. Anatomical site, typing, virulence gene profiling, antimicrobial susceptibility and resistance genes of *Streptococcus suis* isolates recovered from pigs in Spain. *Antibiotics.*  2021;10:707. doi:10.3390/antibiotics10060707.

14. Queenan K, Häsler B, Rushton J. A One Health approach to antimicrobial resistance surveillance: is there a business case for it? *Int J Antimicrob Agents.*  2016;48:422-7. doi:10.1016/j.ijantimicag.2016.06.014.

15. Resapath. French surveillance network for antimicrobial resistance in bacteria from diseased animals. Annual report. 2020.

16. Ribeiro SO, Fraselle S, Baudoux D, et al. Proposals for antimicrobial testing guidelines applied on ajowan and Spanish lavender essential oils. *Planta Med.*  2021;87:754-63. doi:10.1055/a-1475-0020.

17. Hall CW, Mah TF. Molecular mechanisms of biofilm-based antibiotic resistance and tolerance in pathogenic bacteria. *FEMS Microbiol Rev.*  2017;41:276-301. doi:10.1093/femsre/fux010

18. Costerton JW, Lewandowski Z, Caldwell DE, et al. Microbial biofilms. *Annu Rev Microbiol.*  1995;49:711-45. doi:10.1146/annurev.mi.49.100195.003431

19. Uruén C, Chopo-Escuin G, Tommassen J, et al. Biofilms as promoters of bacterial antibiotic resistance and tolerance. *Antibiotics.*  2021;10:3. doi:10.3390/antibiotics100

20. Grenier D, Grignon L, Gottschalk M. Characterisation of biofilm formation by a *Streptococcus suis* meningitis isolate. *Vet J.*  2009;179:292-5. doi:10.1016/j.tvjl.2007.09.005

21. Bonifait L, Grignon L, Grenier D. Fibrinogen induces biofilm formation by *Streptococcus suis* and enhances its antibiotic resistance. *Appl Environ Microbiol.*  2008;74:4969-72. doi:10.1128/AEM.00558-08.
